# An Ongoing Gender Revolution in Europe: Women’s Stable Employment as a Precondition for Partnered First Births

**DOI:** 10.1007/s11113-026-09990-6

**Published:** 2026-02-09

**Authors:** Angela Greulich, Michael S. Rendall

**Affiliations:** 1 Centre de Recherche Sur Les Inégalités Sociales (CRIS), Sciences Po, Paris, France; 2 Institut Universitaire de France, Paris, France; 3 Department of Sociology and Maryland Population Research Center, University of Maryland, College Park, USA

**Keywords:** Fertility, Female employment, European comparison, Education, Gender roles

## Abstract

The literature on the micro-level gendered associations between employment and fertility in couples has presented a mixed picture, contrasting a uniformly positive association of employment and first birth for men with negative, zero, or positive associations for women. Differences in period, country context, and women’s educational level have been proposed as explanations for the ambiguous findings. We attempted to resolve these differences and explanations by estimating the employment associations for co-residential different-sex couples’ first birth in 24 European countries using the 2004–2017 waves of the European Union Statistics on Income and Living Conditions (EU-SILC) panel survey. We paid particular attention to the stability of women’s pre-conception employment. We found that having both the woman and man full-time, full-year employed was associated with a higher first-birth risk relative to only the man full-time, full-year employed (“male-breadwinner”) and relative to neither the woman nor the man full-time, full-year employed. Women’s full-time, full-year employment across two pre-conception years was strongly positively associated with the risk of first birth for women’s low-, medium-, and high-educational-attainment groups. The association of women’s full-time, full-year employment with first birth was positive not only overall, but also separately for Western-, Eastern-, and Southern-European country groups. These findings suggest that women’s stable full-time employment may be a general precondition for initiating parenthood among European couples.

**Supplementary Information:**

The online version contains supplementary material available at10.1007/s11113-026-09990-6.

## Introduction

A revolution in family and employment gender roles in high-income countries has been underway for decades, although it has progressed at different paces and along uncertain trajectories across countries (Cotter et al., 2011; England, 2010; Esping-Andersen & Billari, 2015; Goldin, 2006; Knight & Brinton, 2017). At the aggregate level, higher levels of women’s labor force participation have become associated with higher fertility levels in high-income countries in recent decades (Ahn & Mira, 2002; Doepke et al., 2023; Engelhardt et al., 2004; Oshio, 2019). At the behavioral level within high-income countries, however, the association between women’s employment and fertility appears less clear. Findings from analyses of individual- and couple-level behavior with respect to women’s employment and fertility in Europe have been highly mixed. In particular, studies using standard observational research designs that considered women’s and men’s employment statuses shortly before a birth have often shown negative or zero associations between women’s employment status and fertility (Andersson et al., 2014; Dantis & Rizzi, 2020; Matysiak & Vignoli, 2013; Pailhé & Solaz, 2012; Schmitt, 2012; Vignoli et al., 2012). More recent studies, however, have increasingly found that women’s employment and fertility are positively associated (Kristensen & Lappegård, 2022; Scherer & Brini, 2023; Schmitt, 2021; van Wijk et al., 2022; Zhou & Kan, 2019; but see also Hsu, 2023).

Observational study designs that include comparisons across larger numbers of European countries have generally observed women’s employment status only at a single point in time in the year before birth exposure (González & Jurado-Guerrero, 2006; Adsera, 2011; Hsu, 2023). A single point in time may not adequately capture the potentially important role of employment stability—or, conversely, employment uncertainty—for fertility decisions (Buh, 2023). Moreover, when this single observation occurs in the year immediately preceding the birth or birth exposure, endogeneity between employment and fertility decisions poses a potential threat (Wooden et al., 2023).

In this study, we used extended pre-birth employment observation to define “stable employment,” and we applied this definition across 24 European countries to estimate the association between women’s stable full-time employment and partnered first birth, taking into account the joint employment statuses of partners in co-residential different-sex couples. We drew on a harmonized panel-survey database, the European Union Statistics on Income and Living Conditions (EU-SILC), for the years 2004–2017. Our ability to observe women’s full-time, full-year employment status in up to two pre-conception years was enabled by the application of multiple imputation for left-censored employment histories, thereby overcoming EU-SILC’s limitation of only four annual panel waves per sampled household.

We also examined whether women’s stable employment can be considered a general precondition for first birth across European countries or whether the association between women’s stable full-time employment and partnered first birth varies across European regions and across education groups within countries. The EU-SILC data encompass all major regions of Europe and therefore capture varied family-policy and normative contexts. Prior studies have found that the positive employment-fertility association for women may be limited to, or stronger in, the more gender-equal countries especially in Northern Europe. We therefore investigate the association of women’s stable full-time employment with partnered first birth not only overall, but also separately for Southern, Eastern, and Western European country groups. Prior studies have also found that only or mainly high-educated women have a positive employment association with first-birth propensities, and therefore we investigate separately the employment-first birth associations for low-, medium-, and high-education groups.

## Background

### Gendered Theory of Employment and Fertility

A mix of economic and sociological theory has informed studies of the association between women’s and men’s employment and couple fertility. In economic studies, the theory of employment effects on fertility has commonly been placed in a framework of positive income effects and negative substitution effects (Ayllón, 2019; Bellido & Marcén, 2019; Huttunen & Kellokumpu, 2016). Schaller (2016) referred to this as “the Becker model” (Becker, 1960) and described its main predictions as men’s earnings having an unambiguously positive “income effect” on fertility (more strictly, on “total demand for children,” to reflect both child quantity and child quality), whereas women’s earnings have an ambiguous direction because the positive income effect may be offset by a negative “substitution effect” in which children have an “opportunity cost” on women’s time that they could, without children, spend in paid employment. As Schaller (2016, p. 4) prefaced, this is “Assuming that females are the primary caregivers….”

Sociological approaches have both challenged the assumed primary-childcarer role of the woman in the couple and have studied how differences in the norms for childcare responsibilities across different societies influence gender differences in employment and associated earnings effects on fertility. Influential work by Oppenheimer (1988) and Sweeney (2002) has examined changes over time in the United States in norms and couple expectations for women’s and men’s respective contributions to family income through employment, and to family wellbeing more generally through childcare and other family and household tasks. Knight and Brinton (2017) have documented similar movements of social attitudes away from traditional gender roles on employment and family across European countries during the 2000s, while Wielers and Raven (2013) have documented reductions in both men’s and women’s employed hours to facilitate their contributions to childrearing. Brinton and Lee (2016) examined differences in these gender ideologies for employment and family roles in a couple across high-income countries and their consequences for gender differences in employment and fertility. They referred to the Becker model as embodying a “gender-essentialist norm that women are the natural caregivers” (p. 424). The employment-role corollary of this gender-essentialist norm is the “male breadwinner” model. In Europe as in the United States, however, this model has been challenged as being no longer an accurate description of either social norms or empirical patterns (Goldin, 2006; Goldscheider et al., 2015; Knight & Brinton, 2017; Olivetti, 2006).

### Women’s Employment Trajectories and Pre-birth Employment Stability

An important development in economic theorizing of women’s employment and fertility has been toward a dynamic characterization in which the woman will be motivated to sequence her employment and fertility events to improve her employment and earnings trajectories during the childrearing years. Del Bono et al., (2012, pp. 660–661) proposed two mechanisms that induce women to base their childbearing decisions on the future employment-trajectory implications of their sequencing of stable employment and first birth. The first mechanism is a negative “employability” effect of having a child while not stably employed, in which employers discriminate against a mother (but not a father) with a young child. They hypothesized that “pregnant women or women with small children might be less attractive to potential employers” (p. 661). Huttunen and Kellokumpu (2016, p. 430) similarly noted the importance of this mechanism, concluding that women without employment will defer childbearing because they “…either fear having trouble finding new employment…or they want to secure their careers in new jobs before leaving on maternity leave.”

The second mechanism is a negative “career” effect of a woman’s beginning childbearing very early in a job or employment trajectory, when human-capital formation may be especially important. At stake here is “the loss of future income that is incurred” (Del Bono et al., 2012, p. 661). They proposed that these two dynamic mechanisms related to employment and birth sequencing are more relevant to a woman’s childbearing decision than is the standard one-period model’s “income effect” versus “substitution effect” calculus of whether being employed and having earnings in the current period is positive for childbearing (a positive “income effect” on her fertility), or if being unemployed in the current period is positive for childbearing by reducing the market value of her time (a positive “substitution effect” on her fertility). In single-country studies using quasi-experimental designs incorporating exogenous losses of employment and multiple periods of employment status before the period of birth exposure, Del Bono et al. (2012), Huttunen and Kellokumpu (2016), Hofmann et al. (2017), and Di Nallo and Lipps (2023) all found positive effects of women’s employment on fertility, as inferred from negative effects of job loss. Andersen and Özcan (2021), however, found a negative effects.

Differences in findings across studies, whether they are from quasi-experimental or conventional observational designs, may be due in part to how and when employment status is measured relative to fertility exposure. This is likely to matter more for women than for men given gender differences in disruptions to employment induced by a birth. The use of multi-period longitudinal data crucially allows for the observing of women’s, and their male partner’s, employment not only before the birth but also for one or more years before the conception leading to the birth. This has two advantages. First, as shown by Wooden et al. (2023), observation of employment status only shortly before the birth exposure may induce endogeneity bias resulting from the woman’s (but not the man’s) immediately pre-birth employment status being chosen simultaneously with the birth decision, whereas this is less likely with a longer employment-status lag. Second, longitudinal designs may better capture the concepts of employment instability and uncertainty (Buh, 2023). One frequently used measure of employment instability and uncertainty is whether an employment contract is “temporary” or “permanent,” with temporary contracts typically found to reduce birth propensities (Alderotti et al., 2021; Lass, 2020; Lundström & Andersson, 2012; Scherer & Brini, 2023), although increased birth propensities have also been found (Hsu, 2023). Wooden et al. (2023), however, found a likely endogeneity bias specifically when using this temporary-contract measure. Additionally, contracts have different meanings across different countries, and measures may not be available consistently, or at all, across countries. Kopycka et al. (2023) thus argued instead for using multi-period employment statuses to measure employment instability in cross-national studies.

Due to the heavy data requirements, however, both quasi-experimental and observational studies that are based on longitudinal data that include employment-status observation in multiple years before fertility exposure are often restricted to only one country (Del Bono et al., 2012; Hofmann et al., 2017; Huttunen & Kellokumpu, 2016; Pailhé & Solaz, 2012; Schmitt, 2021; van Wijk et al., 2022), or at best to only two or three countries (Di Nallo & Lipps, 2023; Musick et al., 2020), limiting scope for providing comparative evidence for Europe.

### Regional Variation in the Association of Women’s Employment with Fertility

Egalitarian gender norms and family policies encouraging work/life balance (e.g., formal childcare, remunerated parental leave) may facilitate women’s combining employment with childbearing. Both make it more likely that a woman’s employment will have a predominantly positive “income” effect on fertility, just as it does for men, with negative “substitution” effects for women becoming less applicable (McDonald, 2000; Neyer et al., 2013). Movement toward greater equality between women’s and men’s employment and family roles has differed by European country context (Arpino et al., 2015; Kalmijn, 2013). This reflects variability in family policies and normative environments. Nevertheless, there has been evidence for a pervasive movement of social attitudes away from the male breadwinner model in 21st-century Europe (Knight & Brinton, 2017). Labor-market institutions have also been found to play a role, and these vary across European countries (Adsera, 2011). Ongoing progress toward greater gender equality in European labor markets, however, has been seen in a near-pervasive decline in the gender pay gap across European countries in the 2000s (Kunze, 2018). Time lags in the effects of gender-role attitudinal changes (Arpino et al., 2015) and family-policy changes (Hook & Paek, 2020; McDonald, 2006), however, suggest that the positive employment-fertility association for women may be limited to, or stronger in, the more gender-equal countries of Northern Europe than in the more traditional gender-role countries of Southern Europe, with Western and Eastern Europe somewhere in between (Alderotti et al., 2021).

*Northern Europe* A pattern of “positive or no association” of women’s employment with fertility is the general conclusion from studies of Northern European countries, with positive associations found especially in more recent studies. In Norway, Kravdal (2002) found no association between women’s employment and first birth, but Kristensen and Lappegård (2022) found a positive employment association with first birth for both women and men. In Finland, Miettinen and Jalovaara (2020) found an overall positive association between women’s employment and first birth, but an even larger positive association between men’s employment and first birth. For both women and men, they found the biggest contrast was between the employed and the long-term unemployed, for whom fertility is lowest. In Sweden, positive associations of women’s employment with first birth and men’s employment with first birth were found by Lundström and Andersson (2012). A positive association of women’s employment and first birth was also found in Denmark (Andersen & Özcan, 2021; Andersson et al., 2014; Kreyenfeld & Andersson, 2014).

*Western Europe* Turning to Western Europe, for the Netherlands, van Wijk et al. (2022) found that economic precariousness, measured in trajectories of income levels and activity statuses, decreases the probability of conceiving a first child for both genders, but for men more than for women. For France, which is a country with family policies that support the combining of employment and childrearing for women (Gornick et al., 1997; Thévenon, 2015), Pailhé and Solaz (2012) found no overall association between women’s unemployment and first birth in France through 2005. Schmitt (2012) similarly found no statistically significant association of women’s employment with entry to parenthood when using the 1994–2000 European Community Household Panel (ECHP). Also using the ECHP, however, González and Jurado-Guerrero (2006) found a positive women’s employment association with first birth in France, with the “dual-earner” couple employment configuration of both employed having higher first-birth propensities than “male-breadwinner” configurations of the man employed and the woman either unemployed or inactive. This is complemented by Meron and Widmer’s (2002) findings, using different French data up to 1997, of different associations of employment with first partnered birth according to whether a woman was not employed and out of the labor force (increasing the likelihood of giving birth) versus was unemployed (decreasing the likelihood of giving birth).

*Southern Europe* Findings from studies of Southern European countries, which are viewed as having the least gender equality and the least facilitative family-employment-combination policies and normative contexts (Barbieri et al., 2015), have been largely consistent with the standard theory of men’s employment having a positive effect and women’s employment having a negative effect on fertility. Two studies of Southern European countries used the EU-SILC data: Dantis and Rizzi (2020), using the EU-SILC 2005–2013 for Greece, found that having the man unemployed or inactive reduced the probability of first birth, whereas having the woman unemployed or inactive did not. Vignoli et al. (2012), using the EU-SILC 2004–2007 for Italy, found a similar gender asymmetry in the employment association with first birth. The chance of having a child was higher for unemployed or inactive women than for their employed counterparts, while the pattern for men was the opposite. However, their sample size was too small to draw statistically significant conclusions.

Matysiak and Vignoli (2013) studied an earlier period in Italy, through 2003, and found a clearly negative association of women’s employment with first birth. Alderotti (2022), however, found that the link between women’s employment and transitions to first births was negative in Italy before 2010 but became positive after 2010. Consistent with the latter finding of an emerging positive association of stable women’s employment and first birth, Alderotti et al. (2023) found that in Italy, unemployment and career instability negatively affected women’s probability of motherhood in the aftermath of the Great Recession.

*Eastern Europe* Regarding Eastern European countries, Matysiak and Vignoli (2013) found no association of women’s employment with first birth for Poland, a finding corroborated with more recent data by Hsu (2023). Hsu (2023) found a variety of associations of female temporary employment or unemployment with first-birth propensities, from being negative in Croatia (hence a positive association of stable employment with first birth) to being positive in Latvia, Lithuania, Czech Republic, and Slovakia (hence a negative association of stable employment with first birth). The negative employment-fertility associations in the Czech Republic and Slovakia were further argued by Hsu to be attributable to family-policy conditions that compensate women for negative income effects of withdrawing from the labor force. In summary, there is overall more evidence for zero or negative associations of women’s stable employment with first birth in Eastern Europe in the existing literature.

### Educational Variation in the Association of Women’s Employment with Fertility

In both Europe and the United States, women’s educational attainment has been proposed as a major additional axis of differentiation, with higher-educated women more likely to combine employment and family roles, leading to a positive employment-fertility association that is limited to more educated women (Kreyenfeld, 2010; Musick et al., 2020; Yu & Sun, 2018). Several European studies have found that only or mainly high-educated women have a positive employment association with first birth (e.g., Kreyenfeld & Andersson, 2014, for Germany and Denmark; Barbieri et al., 2015, for Italy and Spain). Miettinen and Jalovaara (2020) also concluded that the direction of the employment–first-birth association for women depends on her educational attainment. However, they only found a negative association between employment and first birth for the least-educated women at ages 18–24, whereas at ages 25–30 and 31–39, positive associations between employment and first birth were apparent for women of all four levels of educational attainment.

Sociologists and economists described women with high educational attainments as having more to lose in terms of securing a career with a rising earnings trajectory if they begin childbearing without first having secure employment (Miettinen & Jalovaara, 2020). More educated women have also been presumed in the sociological literature to be more “career minded” (Kreyenfeld, 2010, p. 354) and thus more inclined than less educated women to defer childbearing until they have stable employment. Moreover, Second Demographic Transition theory proposes that those with more education are the first to adopt new family-demographic and family-economic behaviors, of which greater equality between the woman and the man in a couple in emphasizing labor-market achievement is a major component (Lesthaeghe, 2010; Zaidi & Morgan, 2017).

From an economic perspective, Del Bono et al.’s (2012) “career effect” mechanism explicitly differentiates women by their socioeconomic positions in ways that may be analogous to education effects. Empirically, Del Bono et al. differentiated between women in white-collar occupations and blue-collar occupations and found a positive employment association with fertility for women with white-collar career trajectories. This was reflected in a large negative effect of job loss on fertility for women in white-collar occupations but not for women in blue-collar occupations.

An alternative theoretical perspective, however, is that lower-socioeconomic-status couples may be less able to put at risk potential future income from the woman’s earnings and therefore would prioritize pre-birth employment at least as much as would higher-SES women. Ayllón (2019) adopted this perspective in predicting and finding with aggregate data on unemployment rates that less-educated women are more sensitive to the negative effect of overall unemployment on their fertility than are more-educated women. Meron and Widmer (2002), using individual employment-status data, similarly found a negative effect of unemployment on first birth for less-educated women of a magnitude that is at least as great or greater than that for more-educated women.

### The Current Study

Given the mixed picture of employment-first birth associations currently in the literature, with differences across country groups and by women’s educational attainment in particular, and with potential endogeneity concerns for the measurement or women’s pre-birth employment, the current study aims to provide greater clarity by investigating partnered first birth in 24 European countries for the years 2004–2017, using EU SILC panel data. These data encompass all geographical regions of Europe, representing varied family policy and normative contexts.

We used country and year fixed-effects logistic regression models to estimate associations of partnered first births with full-time, full-year employment statuses of women observed two and three years before a first-birth exposure year, and of both couple members observed two years before first-birth exposure. Despite the general limitation of the EU SILC to only four annual panel waves per sampled household, we were able to include sufficiently large numbers of couple observations of first-birth exposure in part by employing multiple imputation for left-censored trajectories (Rendall & Greulich, 2016). We modelled the stable-employment and first-birth association across three geographical-area country groups and across three levels of women’s educational attainment. We thereby aimed to re-evaluate the association between women’s employment and partnered first births in Europe based on: (1) the largest possible country and time coverage; (2) observed employment duration that is sufficiently long to capture ‘employment stability’; (3) sufficient time delay between employment and first birth to minimize endogeneity bias; and (4) with consideration of potential interactions partner employment, country context, and women’s educational attainment.

Drawing on the gender-revolution framework, we suggest that due to changes in gender-roles across socio-economic contexts, woman’s employment stability may have become a general critical precondition for first childbirth in Europe and beyond. We therefore propose and test hypotheses of three types describing the association between stable employment and partnered women’s and men’s first-birth propensities:

1. *Gender hypothesis*: the couple’s first-birth propensity is positively associated not only with the man’s, but also with the woman’s, stable employment.
2. *Country gender-equality, labor-market institutions, and family-policy context hypothesis*: first-birth propensities are positively associated with the woman’s stable employment across country contexts.
3. *Woman’s educational attainment hypothesis*: first-birth propensities are increased by the woman’s stable employment for women across all education levels.

## Data and Methods

Our data were drawn from the 2004–2017 waves of the European Union’s Statistics of Income and Living Conditions (EU SILC) panel survey (Eurostat, 2023), covering the large majority of European countries. EU-SILC captures individual and household situations by providing a set of harmonized economic and social variables that may be considered as determinants for fertility decisions (labor force status and education in particular), and it provides a multi-wave annual follow-up of individuals and their households. Using this longitudinal sample allowed us to ensure a sufficient time delay between observation of labor-force statuses and first-birth exposures. The standard longitudinal implementation of the EU-SILC consists of a rotational panel in which individuals are observed annually for a period of four years (i.e., four waves).^1^ Selection into the sample occurs annually, beginning in 2004, and each new sample after 2004 is followed for four waves,^2^ with households and individuals aged 16 and above followed in the case of a move or a household dissolution, and with generally high rates of wave-to-wave follow-up. Iacovou et al. (2012) reported a median 87% individual follow-up response rate from one wave to the next in the 2005–2008 EU-SILC waves. They also noted, however, heterogeneity across countries in their success in following household and individual’s moves, notwithstanding the uniform requirements of Eurostat with respect to following rules (see also Eurostat, 2012).

Our analytical sample covers 24 European countries.^3^ Although the EU-SILC surveys 32 countries, some countries were excluded from our analyses due to data privacy or non-conforming protocols (e.g., Germany), data-harmonization issues (e.g., Norway), and fertility measurement incompatibility (e.g., Romania). Section A in the online supplement provides more details on country exclusions. For about half of the countries, the first panel samples started in 2004, and for the other half they started in 2005 (except for Switzerland which the first panel sample started in 2011).

### Sample Selection

**Fig. 1:**
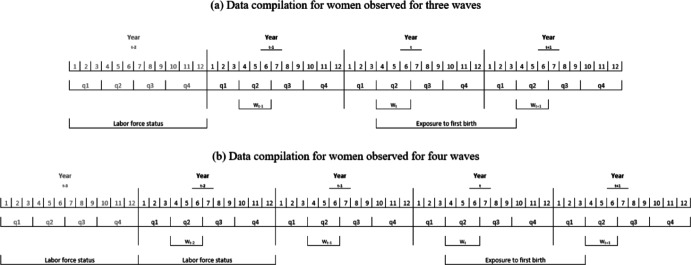
Illustration of the coding of full-time, full-year employment in year t−2 and year t−3 to predict the probability of first birth between w_t_ and w_t+1_. *Notes:* The first-birth event variable is coded 1 if a first child was born in one of the quarters between w_t_ and w_*t*+1_. In this example, both the interviews at waves w_t_ and w_*t*+1_ take place in the second quarter of each year, i.e., in April, May, or June. The interviews of waves w_t_ and w_*t*+1_ identify births that occurred between these two interview waves. The observed period of exposure to first birth is from quarter 2 (q2) of calendar year t up to but not including the interview occurring in quarter 2 (q2) of calendar year t + 1. For 3 out of 4 women in our sample, interviews take place in either the first or second quarter of the year, and in the same quarter of each survey year. Year_t−2_ is the 12-month period two years before the year of beginning first-birth exposure (which is in year t, at wave w_t_, beginning the exposure year from w_t_ to w_*t*+1_). Year_t−3_ employment status, the 12-month period three years before the year of beginning first-birth exposure, is either observed (see **b**) or is multiply-imputed when missing due to left-censoring (see **a**). **a** Also illustrates the treatment of the woman’s left-censored-employment year of exposure to first birth before her fully-observed employment year of exposure to first birth. This will be the case when she does not have a birth in the first, left-censored-employment year that is observed in the EU SILC household record

We limited our analyses to women aged 18–39 and who lived in co-residential different-sex couples (either married or cohabiting) and were childless in the year before the year of first-birth exposure (i.e. at w_*t*+1_, see Fig. 1)_._ The upper age restriction to 39 is needed so that we could reasonably approximate a woman being of parity 0 from the household variable of her having no co-resident children.^4^ The focus on co-residential women allowed us to apply a couple rather than an individual perspective of the nexus between employment configurations and first childbirth. We additionally restricted our analyses to women who continue to live with the *same* male partner through the year of their first birth exposure (observed at w_t_) to reduce fertility-linked attrition bias caused by partnership dissolution. Furthermore, to observe labor-force status during at least one full calendar year before potential conception of a first child, a minimum of three consecutive waves was required. This allowed us to proxy employment stability, as well as to guarantee a time delay between employment and potential conception. The latter is important to minimize endogeneity between employment and birth decisions.

Figure 1 illustrates our data compilation procedure for three-wave cases (Fig. 1a) and four-wave cases (Fig. 1b). In Fig. 1a, the three interview waves are designated w_*t*−1_, w_*t*_, and w_*t*+1_, occurring in calendar years that we designate as *t−1*, *t*, and *t* + *1*. For women who remained childless until w_*t*_, exposure to first birth was observed between w_*t*_ and w_*t*+1_, and labor force status was observed on a monthly basis during the full calendar year *t−2* (i.e. a 12-month period)*.* For four-wave cases, we could observe full-time, full-year employment over a 24-month period (Fig. 1b). More detailed information about the coding of first births and labor force status is provided in the ‘Measures’ subsection below.

We excluded from our analytic sample women who were students or in further training at w_*t*_ (at the beginning of exposure to first birth), which allowed us to better focus on the employment-fertility nexus. To observe not only women’s but also their male partner’s employment during at least one full calendar year before potential conception of a first child, women must have been continuously partnered for at least two waves (w_*t*−1_ and w_*t*_). These restrictions made the women in the population we analysed somewhat more educated than the overall population of partnered women exposed to first birth. Accordingly, they reduce the generalizability of our study to relatively stable co-residential different-sex couples. Section B in the online supplement provides more details on sample selection.

Our analytical sample contained 12,060 person-year observations. The restriction to birth-exposure intervals after having observed labor-force status at least as far back as year *t−2* implied a considerable reduction in sample size (as quantified in Table S1 in the online supplement). This resulted in our being able to analyze cross-country differences only by forming country groups that represent broad geographical regions. The crucial gains from extending the observed time period for employment, however, were both that this addressed the potential endogeneity of employment status with conception and birth, and that it allowed us to better capture employment stability, specifically as full-time, full-year employment over a 12-month period and over a 24-month period. Section C in the online supplement provides further information about the advantage of our measure of employment stability compared to alternative indicators such as labor-force status observed at the time of the survey and employment contract type.

### Measures

*Dependent Variable, First Births* To identify first births in EU-SILC, we applied the “own-children method” (Rindfuss, 1976), which consists of merging household members to each other and in linking a woman to her co-resident children, including newborns. The household merge also allowed the linking of the woman to her co-resident partner and thereby enabled observation of that partner’s characteristics. The household merge was possible as in EU-SILC every household member had a register file with basic demographic information, and the EU-SILC’s variable for year and quarter of birth allowed us to identify newborn infants. We identified parity (in our case, nulliparous couples) by considering children who are living in the observed household at the time of the survey. The couple was defined as nulliparous if there was no child in their household observed before the quarter of the interview of year *t* (designated as w_*t*_, see Fig. 1). We identified births to couples from observing infant children who were new to the household since the previous survey wave.

Identifying births with the “own-children method” was necessary because births were not observed directly in the EU-SILC. There was no variable in the database for the current number of children living in and outside the household or for children ever born. Nor was there a variable for whether a child was born in the last year. Measuring fertility with the “own-children” method carries the risk of obtaining fertility measures that are somewhat biased, mainly due to underreporting and attrition. Greulich and Dasré (2017) have quantified these biases comprehensively for all countries in EU-SILC and found that the bias remains small for the large majority of countries. Furthermore, they showed that econometric analyses of socio-economic determinants of transitions to births were unlikely to be affected by biases in the measurement of fertility. Section D in the online supplement provides further details on potential biases in the measurement of fertility in EU-SILC.

Our outcome *first-birth event* variable was coded 1 if a linked child was born in one of the quarters between w_t_ and w_*t*+1_ for women who remained childless until w_*t*_*.* In the example illustrated in Fig. 1a, both the interviews at waves w_*t*_ and w_*t*+1_ took place in the second quarter of each year, i.e., in April, May, or June. We identified births that occurred between these two interview waves. The observed period of exposure to first birth in Fig. 1a was from quarter* 2* (*q2*) of calendar year *t* up to but not including the interview occurring in quarter 2 (*q2*) of calendar year *t* + *1*. For 3 out of 4 women in our sample, interviews took place in either the first or second quarter of the year and interviews took place in the same quarter of each survey year.

*Independent Variable, Employment* We used the full-year reporting of employment in the EU-SILC^5^ to measure our main employment predictor variable of *full-time, full-year employment* over a calendar year (abbreviated as “*FT employment* “ in the estimation results- sections). Labor-force status was reported on a monthly basis during the full calendar year *preceding* the interview wave. This included the calendar year prior to the first wave. This is shown in Fig. 1a as calendar year *t−2*, as reported in interview-wave w_*t*−*1*_. The time between the last month of observed labor-force status and the beginning of exposure to a birth was therefore a minimum of 12 months and a maximum of 21 months (as defined in quarters, as we only knew the quarter of interview). In our analytical sample, for 3 out of 4 individuals, this time interval was either 12 or 15 months. With this minimum delay of 12 months, we excluded the possibility that a woman was already pregnant with her first child by the most recent month of observation of her labor-force status (year *t−2*). To observe labor-force status during *two* full calendar years before potential conception of a first child, women had to be observed for four annual waves (see Fig. 1b). For women who remained childless until w_t_*,* we observed their labor-force status throughout all 12 months of calendar year *t−2* and all 12 months of calendar year *t−3*. Information on monthly labor-force status in year *t−2* was reported at *w*_*t−1*_ and information on monthly labor-force status in year *t−3* was reported at *w*_*t*−2_. This was considered the ‘completely-observed’ case because 24 consecutive months of labor-force status before potential conception of a first child were observed. Of the 12,060 person-years of first-birth exposure, there were 4,431 ‘completely-observed’ cases.

Following Rendall and Greulich (2016), we used multiple imputation (MI) for left-censored data to retain the same sample sizes for our *t−3* and *t−2* employment-status models as for our *t−2*-only employment-status models. This meant that for the ‘incomplete’ cases in which observation sequences were of only three waves, as was the case for the first three years of a four-year observation that included first-birth exposure in both the third and fourth EU SILC panel waves, the woman’s prior year’s (*t−3*) employment was imputed from the ‘complete’ cases of observed sequences of four waves. Section E in the online supplement provides more detailed information about our MI procedure.

*Control Variables* We controlled for women’s *age* and *marital status* throughout our regression analyses, and we either controlled for, or stratified by, woman’s *education*. All control variables were observed in the interview wave that marked the beginning of birth exposure interval (w_t_ in Fig. 1). Age was included as categorial variable. Education was divided into three levels: *low* (pre-primary, primary, lower secondary), *medium* (upper-secondary, post-secondary non tertiary) and *high* (tertiary).^6^ We included employment and education but not income among the regressors, consistent with our trajectory-based theoretical approach. The findings of Del Bono et al. (2012) and Huttunen and Kellokumpu (2016) indicated that it is not the current-period income losses associated with being without a job that are the important characteristics of employment or employment loss, but instead that it is the employment-trajectory mechanism that is crucial, through its effects on permanent income. Therefore employment stability, and potentially educational attainment, were the critical variables to include among the predictors.^7^

### Empirical Setup

We used a discrete-time binary logit regression model in which the outcome is a birth (versus no birth) observed over a one-year interval. The underlying unobserved propensity for woman *i* in country *j* to have a first birth (*FirstBirth*) was modelled as a function of the woman’s and her partner’s employment status (*E*^*f*^*, E*^*m*^), plus a set of individual covariates (*X*^*f*^_*ij*_) for the woman. Specifying *C*_*j*_ as a country dummy and *T* as a year dummy, we specified a binary logit model with country and year fixed-effects:

$$ FirstBirth_{ij} = \alpha + \beta_{1} \times E_{ij}^{f} + \beta_{2} \times E_{ij}^{m} + \beta_{3} \times X_{ij}^{f} + C_{j} + T \sim { }Logistic\left( {0,1} \right) $$

As some women (couples) in our sample were observed twice (those who were observed for four waves and remained childless until w_t_), we ran regression models with cluster-robust standard errors.

The employment-status variables *E*^*f*^ and *E*^*m*^ differed across our estimation models as follows. For our analyses focusing on couple combinations of labor-force statuses, *E*^*f*^ and *E*^*m*^ were defined for the 12-month period two years (year_t−2_) before the year of beginning exposure to first birth. We alternately used a four-category and an eight-category variable representing the types of couple combinations of labor-force statuses. For our more detailed analyses of the women’s own employment stability, we used *full-time, full-year employment (“FT employment”*) defined for the 12-month period two years (year_t−2_) and additionally for the 12-month period three years (year_t−3_) before the year of beginning exposure to first birth.

We additionally estimated our logistic model separately for samples of women in each of the three education groups (high, medium, and low), and separately for women in the three largest-sample regional groups (Western, Eastern, and Southern Europe^8^). To test for whether the employment-stability contrast differed across educational attainment groups, we ran a regression model pooling the three education-level samples and used difference-in-difference in probabilities tests for interactions between educational attainment and our four-category variable of woman’s stability of employment. We similarly tested for interactions between the country group variable and our four-category variable of full-time, full-year employment using difference-in-difference in probabilities, again running a pooled regression model, and in this case testing for interactions between regions and our four-category variable of couple employment configurations.^9^ Finally, sample weights were used for the descriptive statistics, but not for the regression analyses. Regression results were robust to the inclusion of weights. Unweighted regression models were preferred for presentation as weighting schemes risked not being completely harmonized across all countries in EU-SILC and unweighted regression models yielded somewhat higher statistical efficiency. Women with missing or zero sample weights (a negligible number) were dropped from the analyses.

## Results

### Descriptives

Table 1 provides descriptive statistics for the labor-force status and control variables that were used in the regression models presented in this paper. We classified into a two-by-two grouping the woman’s and the man’s *full-time, full-year employment* observed during the calendar year prior to the year of potential conception of a first child (in year *t−2*). Couples in which both the woman and the man were *full-time, full-year employed* comprised 57.3% of all couples, whereas in 10.1% of couples, neither the woman nor the man was f*ull-time, full-year employed*. For 24.9% of couples, only the male partner was *full-time, full-year employed*, and in 7.7% of couples only the woman was *full-time, full-year employed*. A further disaggregation of couple labor-force statuses observed in year *t−2* shows that 8.7% of couples were both *full-year employed*, but either one or both were *employed part-time* throughout the year. Additionally, 9.8% of women, but only 3.9% of men, experienced *unemployment* in at least one month of the year when their partner was f*ull-time, full-year employed*, and a further 5.0% of women were *inactive* in at least one month of the year (without ever being *unemployed*), versus only 0.3% of men. That is, experiencing *unemployment* or an *inactive l*abor-market status occurred for 14.8% of women, but only 4.2% of men, when their partner was f*ull-time, full-year employed*. Concerning our two-year measure of women’s f*ull-time, full-year employment* (observed in years *t−2* and *t−3*), 56.2% of women were *full-time, full-year employed* in both years, 28.1% were not *full-time, full-year employed* in either year, 9.5% were *full-time, full-year employed* in only the more recent of the two years, and 6.2% were *full-time, full-year employed* in only the earlier of the two years.

**Table 1:** Women’s and male partner’s employment statuses and women’s socio-demographic characteristics, childless women aged 18–39 with a co-resident male partner (weighted statistics)

*Employment-status variables*^a^:	
Combinations of couple full-time, full-year employment	100%:
Only male partner full-time, full-year employed in year_t−2_	24.9%
Only woman full-time, full-year employed in year_t−2_	7.7%
Both full-time, full-year employed in year_t−2_	57.3%
Neither full-time, full-year employed in year_t−2_	10.1%
Detailed couple employment-status and labor-force activity combinations	100%:
Both full-time, full-year employed in year_t−2_	57.3%
Both full-year employed, either or both part-time in year_t−2_	8.7%
Woman full-time, full year; male partner ever unemployed in year_t−2_	3.9%
Woman full-time, full year; male partner ever inactive (never unemployed) in year_t−2_	0.3%
Male partner full-time, full year; woman ever unemployed in year_t−2_	9.8%
Male partner full-time, full year; woman ever inactive (never unemployed) in year_t−2_	5.0%
Either or both ever students in year_t−2_	2.3%
Either full-time, full-year employed; the other changed activity status in year_t−2_	3.3%
Any other combination of neither full-time, full-year employed in year_t−2_	9.3%
Woman's full-time, full-year employment status during the two calendar years prior to the year of potential conception (at years t−2 and t−3)	100%:
Woman full-time, full-year employed in year_t−2_ and year_t−3_	56.2%
Woman full-time, full-year employed in year_t−2_ but not in year_t−3_	9.5%
Woman full-time, full-year employed in year_t−3_ but not in year_t−2_	6.2%
Woman not full-time, full-year employed in year_t−2_ or year_t−3_	28.1%
*Other variables*^b^:	
Educational Attainment	100%:
Low (pre-primary, primary, lower secondary)	11.4%
Medium (upper-secondary, post-secondary non tertiary)	37.8%
High (tertiary)	50.8%
Married	58.1%
Age	31 (median)
N of person-year observations: 12,060^c^	
N of respondents: 8386^d^	

Data Source: EU-SILC, 24 European countries, 2004–2017

^a^Couple employment status combinations are defined for the 12-month period two years (year_t−2_) before the year of exposure to first birth for both women and their male partners; woman’s employment status is additionally defined for the combination of two (year_t−2_) and three (year_t−3_) years before the year of exposure to first birth. (See Fig. 1.)

^b^Women’s education, age, and marital status are defined at the interview Year t (w_t_ in Fig. 1)

^c^N of observations = 12,060 for year_t−2_ employment statuses and for ‘Other variables’; N of observations = 4431 for women’s observed year_t−2_ and year_t−3_ combination of employment statuses

^d^N of respondents = 8386 for year_t−2_ employment statuses and for ‘Other variables’; N of respondents = 4431 for women’s observed year_t−2_ and year_t−3_ combination of employment statuses

Half (50.8%) of women were *higher-educated* (tertiary qualifications), with 37.8% and 11.4% respectively *medium- and low-educated*. Just over half of the couples (58.1%) were *married*, the remainder being in a non-marital cohabitation relationship. The median *age* of the women was 31.

### Regression Models of First Birth on Combinations of Women’s and Men’s Pre-birth Employment Stability

**Fig. 2:**
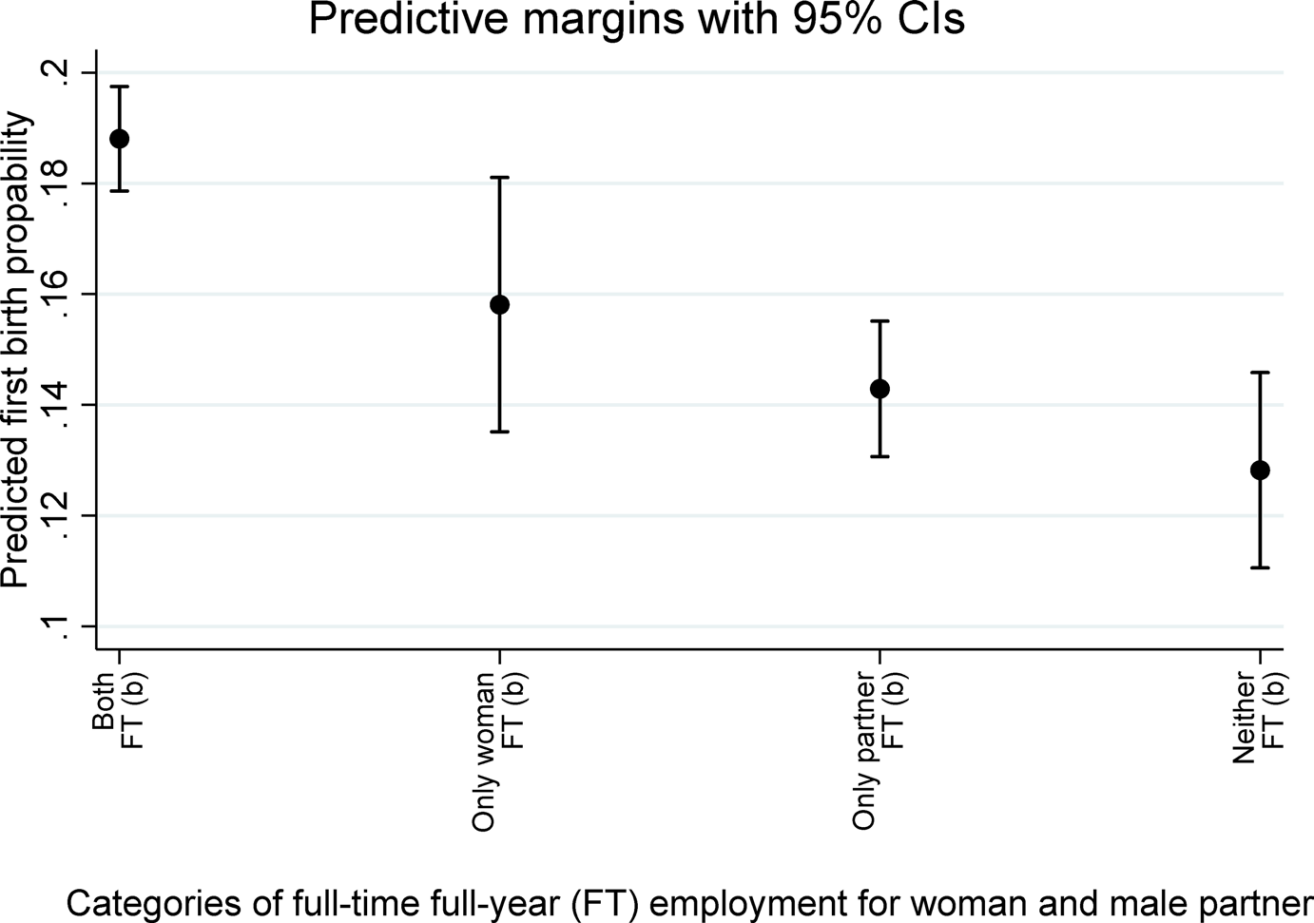
Predicted probability of first birth by woman’s and male partner’s full-time, full-year employment two years before the year of beginning first-birth exposure. Data Source: EU-SILC, 24 European countries, 2004–2017. *Notes:* Predicted first-birth probabilities (at means of covariates) shown are based on regression of first birth on woman’s and male partner’s full-time full-year employment combination, with control variables for marital status and age categories and with country- and year-fixed effects (underlying estimates presented in Table B, column 1, in the online supplement). FT: full-time full-year employment. Categories that do not share a script (a, b) are statistically different from each other at the 95% confidence level. First-birth exposure starts in Year t, and continues into Year t + 1 (see Fig. 1). Full-time, full-year employment status for both the woman and her male partner is defined for the 12-month period two years (year_t−2_) before the year of beginning exposure to first birth (see again Fig. 1)

We first considered evidence that relates to our “gender hypothesis” stating that couple’s first-birth propensity is positively associated not only with the man’s, but also with the woman’s, stable employment. In Fig. 2, we present predicted probabilities of first birth by joint combinations of woman’s and male partner’s *full-time, full-year employment* (“*FT employment*”) in year *t−2*, derived from regression models including controls for *marital status* (married versus cohabiting) and categorical *age*, and including both *year* and *country* fixed-effects.^10^ The underlying estimates can be found in Table S2, column 1, in the online supplement. Figure 2 illustrates predictive margins (at means of co-variates) with 95% confidence intervals. The configuration which had the highest predicted first-birth probability (0.188) is ‘*both partners FT employed*’, whereas ‘*neither FT employed*’ had the lowest first-birth probability (0.128). The configuration of ‘*only the woman FT employed*’ had a predicted first-birth probability of 0.158, whereas the configuration of ‘*only the male partner FT employed*’ had a predicted first-birth probability of 0.143.

Furthermore, compared to the *woman and her male partner both being FT employed*, we found that each of the two configurations in which the *woman was not FT employed* (categories 3 and 4 in Fig. 2) was associated with a statistically-significantly lower first-birth probability. The configuration in which *only the woman was FT employed* (category 2), however, also had a statistically-significantly smaller estimated first-birth probability in comparison to the configuration in which the w*oman and her male partner were both FT employed*.^11^ The latter configuration therefore dominated all others in its being associated with the highest first-birth propensity.

**Fig. 3:**
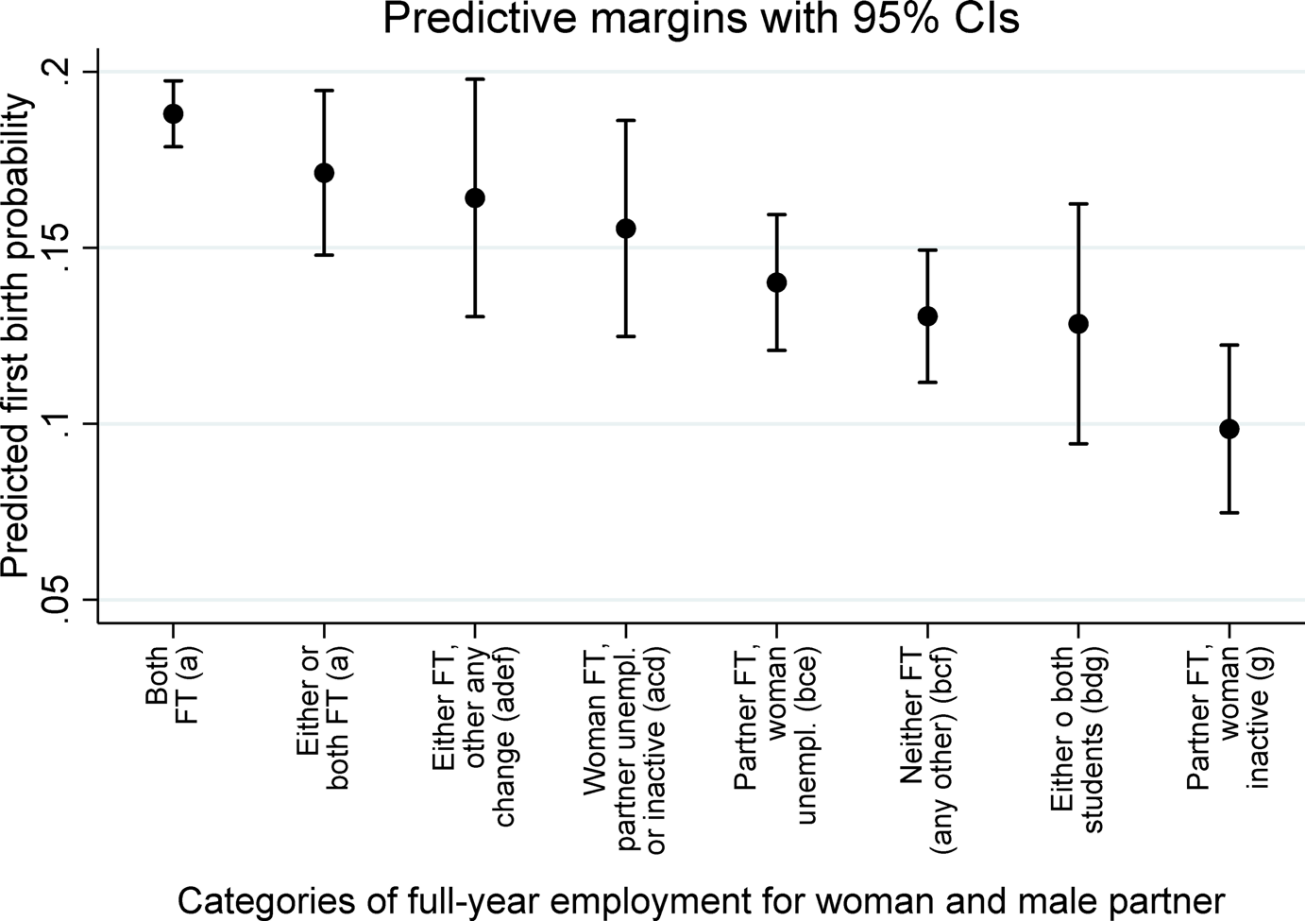
Predicted probability of first birth by couple combinations of full-year employment two years before the year of beginning first-birth exposure. Data Source: EU-SILC, 24 European countries, 2004–2017. *Notes:* Predicted first-birth probabilities (at means of covariates) shown are based on regression of first birth on woman’s and male partner’s detailed employment combination shown, with control variables for marital status and age categories and with country- and year-fixed effects (underlying estimates presented in Table B, column 3, in the online supplement). FT: full-time full-year employment; PT: part-time full-year employment. Categories that do not share a script (a, b, c, d, e, f, g) are statistically different from each other at the 95% confidence level. First-birth exposure starts in Year t, and continues into Year t + 1 (see Fig. 1). Labor-force statuses of both the woman and her male partner are defined for the 12-month period two years (year_t−2_) before the year of beginning exposure to first birth (see again Fig. 1)

Figure 3 illustrates predicted first-birth probabilities from a more detailed disaggregation of eight couple combinations of employment statuses and other labor-market activity statuses. The underlying estimates can be found in Table S2, column 3, in the online supplement. Figure 3 shows that the highest predicted first-birth probability was here again obtained for the first category in which ‘*both the woman and her male partner are FT employed*’ (0.188). The first-birth probability for full-year employment for both partners, with *either or both part-time* (PT, category 2), was not statistically-significantly different from full-time employment for both partners. The category for within-year switches between part-time and full-time employment for one partner, while the other was constantly FT employed (category 3), was also not statistically-significantly different from that for continuous FT employment for both partners.

Of particular interest are couples for whom one was *full-time, full-year employed (FT employed)* while the other spent at least some of the year *unemployed* or *inactive*. We show ‘*unemployed*’ and ‘*inactive*’ separately for the female partner, but combine them for the male partner since very few male partners of FT employed women are ‘*inactive*’. For couples in which the woman is either *unemployed* or *inactive* (while her male partner is *FT employed*), the predicted first-birth probabilities were both substantially and statistically-significantly smaller, at 0.140 for woman-*unemployed* and as low as 0.099 for woman-*inactive*, relative to the 0.188 predicted first-birth probability for couples in which both partners were *FT employed*. Couples in which the male partner was *unemployed* or *inactive* (while his female partner was *FT employed*) also had a smaller estimated first birth probability (estimated at 0.155) compared to couples in which ‘both the woman and her male partner were *FT employed*, but the difference was statistically significant only at the .10 level (*p* = 0.062). This suggests a gender difference in the effect of one, but not both, partners having been *unemployed* or *inactive*. Indeed, we found a statistically-significant difference (*p* = 0.004) between ‘*woman FT employed; male partner ever-unemployed or ever-inactive*’ and ‘*male partner FT employed; woman ever inactive (never unemployed)*’ (last category in Fig. 3). That is, the first-birth risk was reduced more by the woman’s being i*nactive* than by the man’s being *unemployed* or *inactive*.

Finally, both the configuration of neither the woman nor her male partner was *FT employed* (not including here those couples in which either or both were *part-time, full-year employed* throughout the year) and the configuration including *students* (either the man or woman or both) had substantially and statistically-significantly lower predicted first birth probabilities (0.131 and 0.128 respectively) compared to couples in which both partners were *FT employed*.

To sum up, Fig. 2 shows that couple configurations with only one partner *full-time, full-year employed* had lower first-birth probabilities than those with both partners *full-time, full-year employed*. Figure 3 shows in addition that various ‘male breadwinner’ configurations (woman *unemployed* or *inactive* while her male partner f*ull-time, full-year employed*) had among the lowest first-birth probabilities of any configuration, and that these configurations had larger negative associations with first birth than did ‘female breadwinner’ configurations. Thus our ‘gender’ hypothesis received strong support.

### Regional Variation in the Association of Women’s Employment with Fertility

We next considered evidence related to our second hypothesis about possible differences by country gender-equality, labor-market institutions, and family-policy contexts. For this, we estimated separate regression models by country region (Western, Southern, and Eastern Europe). Neither an individual country-by-country analysis nor a separate analysis for Northern countries was feasible due to sample size limitations. There was, of course, substantial within-group heterogeneity concerning the contextual and normative setting, but we abstained from considering them here. The purpose of our analysis in this section was to see whether the overall associations between female and male-partner employment and first birth were found across broad European country groups defined by geographic region, or whether there was instead evidence consistent with the positive female employment association with first birth being limited to certain regions within Europe. We used here the same couple combination of *full-time, full-year employment* variable that we used for Fig. 2, except that we now added woman’s *education* as a control variable to the regression model. Within each country group, we maintained our controls for *country* and *year* fixed-effects.

Table 2 shows the estimated coefficients for the regression models of first birth on couple combinations of women’s and men’s employment in year *t−2*, overall and across the three largest-sample country groups (sample sizes all at least 3,500 person-years in the Western, Southern, and Eastern Europe country-groups). ‘*Full-time, full-year employment* for both the woman and her male partner’ was again the reference category. In terms of signs of the coefficients, more similarities than differences were seen between the country groups. Most signs were negative, implying that generally the configuration of both the woman and her male partner *full-time, full-year employed* was more favorable for first birth than when only one partner, or neither partner, was *full-time, full-year employed*. The contrast between ‘both *full-time, full-year employed*’ and ‘neither f*ull-time, full-year employed*’ was statistically significant overall and for each of the Western, Eastern, and Southern-European country groups. We also found statistically-significant contrasts between ‘both *full-time, full-year employed*’ and ‘only male partner *full-time, full-year employed*’ for each of Western, Eastern, and Southern European country groups. That is, first-birth probabilities were found always reduced in the ‘male-breadwinner’ configuration of only the male partner *full-time, full-year employed*, as this association held across all three of these regional contexts. Both the woman’s and the man’s stable employment was positively associated with first-birth probability in all European regions.^12^

**Table 2:** Logistic regression of first birth^a^ for partnered women aged 18–39 by own and male partner’s full-time, full-year employment status, by country-group

	All^b^	Western Europe	Eastern Europe	Southern Europe
*Full-time, full-year employed in year t−2*^c^				
Both	*Ref*	*Ref*	*Ref*	*Ref*
Only woman^d^	− 0.213*	− 0.857***	− 0.00296	0.0765
	(0.0971)	(0.223)	(0.172)	(0.159)
Only male partner	− 0.315***	− 0.449***	− 0.267*	− 0.318**
	(0.0629)	(0.124)	(0.119)	(0.100)
Neither	− 0.431***	− 0.319*	− 0.484**	− 0.429*
	(0.0915)	(0.159)	(0.187)	(0.168)
*Woman's educational attainment*^e^				
Low	*Ref*	*Ref*	*Ref*	*Ref*
Medium	0.289**	0.496*	0.187	0.273*
	(0.102)	(0.253)	(0.278)	(0.132)
High	0.456***	0.494*	0.457	0.460***
	(0.102)	(0.250)	(0.278)	(0.133)
Married^f^	0.790***	0.791***	0.729***	0.834***
	(0.0586)	(0.0990)	(0.106)	(0.120)
*Age*^f^				
18–22	*Ref*	*Ref*	*Ref*	*Ref*
23–27	− 0.255	− 0.120	− 0.00886	− 0.612†
	(0.177)	(0.337)	(0.301)	(0.319)
28–32	− 0.176	− 0.0812	− 0.0933	− 0.520†
	(0.175)	(0.334)	(0.301)	(0.309)
33–39	− 0.755***	− 0.404	− 0.943**	− 1.012**
	(0.177)	(0.339)	(0.310)	(0.310)
Constant	− 2.006***	− 2.390***	− 2.436**	− 1.263***
	(0.240)	(0.473)	(0.724)	(0.360)
N of person-year observations:	12,000	3454	3594	4087
N of respondents:	8343	2469	2335	2674
Pseudo R-squared:	0.044	0.038	0.056	0.048

Data Source: EU-SILC, 24 European countries, 2004–2017

Estimated coefficients with robust standard errors in parentheses; †*p* < 0.10, *p < 0.05, **p < 0.01, ***p < 0.001; all models include country- and year-fixed effects, and standard errors adjust for clustering within couple in the case of multiple observations of first-birth exposure per couple. Magnitudes of differences between estimated probabilities of full-time, full-year employment categories are not statistically different across country-groups with the exception of statistically significant differences between ‘both full-time employed’ versus ‘woman full-time employed, man not full-time employed’ for Western Europe versus Eastern Europe (*p* = 0.004), and for Western Europe versus Southern Europe (*p* = 0.001)

Country groups are:

*Western Europe:* Austria, Belgium, France, Ireland, Netherlands, Switzerland, United Kingdom

*Eastern Europe:* Czech Republic, Estonia, Hungary, Latvia, Lithuania, Poland, Serbia, Slovenia

*Southern Europe:* Cyprus, Greece, Italy, Malta, Portugal, Spain

^a^First-birth exposure starts in Year t, and continues into Year t + 1 (see Fig. 1)

^b^Includes Northern European countries (N = 865, Denmark, Finland, Sweden)

^c^Full-time, full-year employment status for both the woman and her male partner is defined for the 12-month period two years (year_t−2_) before the year t that begins the year of exposure to first birth (see again Fig. 1)

^d^ ‘Only woman’ full-time, full-year employed coefficient is statistically significantly different from ‘only male partner’ full-time, full-year employed coefficient at* p* < 0.05 for Southern Europe (*p* = 0.02)

^e^Educational attainment categories are: low (pre-primary, primary, lower secondary); medium (upper-secondary, post-secondary non-tertiary); high (tertiary). Education is defined at interview Year t

^f^Age and marital status are defined at interview Year t

Tests revealed no statistically-significant differences between regions in the difference in birth probabilities between ‘b*oth full-time, full-year employed*’ versus ‘*neither full-time, full-year employed*’, nor any difference across regions in the contrast between ‘*both full time, full-year employed*’ versus ‘*male partner full-time, full-year employed, woman not full-time, full-year employed*’. The only statistically-significant differences between regions were found in the difference in birth probabilities that contrast ‘*both full-time, full-year employed*’ versus ‘*woman full-time, full-year employed, male partner not full-time, full-year employed*’. Here, the Western Europe country group stood out, with both Western Europe versus Southern Europe and Western Europe versus Eastern Europe statistically significant (*p*-values = 0.001 and 0.004). In the Western Europe group, couples in which only the woman was *full-time, full-year employed* had relatively low birth probabilities (0.091), whereas in Southern and Eastern European countries, birth probabilities for couples *only the woman wa*s *full-time, full-year employed* were of a similar magnitude (0.178 and 0.199 respectively) to those for couples with *both full-time, full-year employed*. The predicted first-birth probability for ‘*only the male partner was full-time, full-year employed*’ was instead very similar between the country groups (0.144 in Southern Europe, 0.145 in Eastern Europe, and 0.130 in Western Europe, results not shown).

In summary, we found no evidence that the positive association between both full-time, full-year employed and first birth was limited to certain regions in Europe. Instead, in all European regions, couples in which both partners were full-time, full-year employed had a higher first birth probability than couples in which neither was full-time, full-year employed, or than couples in which only the man was full-time, full-year employed. These findings therefore support our second hypothesis that first-birth propensities are positively associated with the woman’s stable employment across country contexts.

### Educational Variation in the Association of Women’s Pre-birth Employment Stability with Fertility

Finally, we considered evidence relating to our third hypothesis, that first-birth propensities are increased by the woman’s stable employment for women across all education levels. Regression models of partnered first birth on woman’s employment stability (full-time, full-year employed over 24 months, in years *t−2* and *t−3*), controlling always for the male partner’s employment and other covariates, are shown in Table 3. Women who were *full-time employed in both years* constituted the reference category. In each case, we controlled for men’s employment in year *t−2* but our focus was on the coefficients for women’s two-year employment sequences. In Column 1 (‘all’), pooling across and controlling for women’s educational-attainment, women who were *not full-time, full-year employed in either of the two years t−3 and t−2*, and women who were *full-time, full-year employed only in the earlier of the two years t−3*—that is, women who exited full-time, full-year employment—both were found to have statistically-significantly lower first-birth propensities than women who were *full-time, full-year employed in both of the two years t−3 and t−2*.^13^ In this ‘all women’ model, women’s *education* was controlled for with dummy variables for medium- and high-educated women relative to the reference low-educated category in this model. These coefficients were both positive and statistically significant. The estimated differences in coefficients between low- and high-educated, as well as between medium- and high- and between low- and medium-educated women, were all statistically significant.^14^ Together, they present a picture of a positive association of education with first birth. This is consistent with the findings of previous European studies (e.g., d’Albis et al., 2017).

**Table 3:** Logistic regression of first birth^a^ for partnered women aged 18–39, by two-year sequence of full-time, full-year employment of woman, by woman’s educational attainment

	All	Low-educated	Medium-educated	High-educated
*Woman full-time, full-year employed*^b^				
In year_t−2_ and year_t−3_	*Ref*	*Ref*	*Ref*	*Ref*
In year_t−2_ but not in year_t−3_	− 0.191	− 0.186	− 0.378†	− 0.129
	(0.153)	(0.501)	(0.221)	(0.195)
In year_t−3_ but not in year_t−2_	− 0.268*	− 0.517	− 0.303	− 0.175
	(0.129)	(0.383)	(0.233)	(0.192)
Neither in year_t−2_ nor in year_t−3_	− 0.343***	− 0.716*	− 0.341**	− 0.263*
	(0.0703)	(0.300)	(0.111)	(0.103)
Partner full-time, full-year employed in year_t−2_	0.155*	0.0518	0.186†	0.156
	(0.0702)	(0.219)	(0.112)	(0.0996)
*Woman's educational attainment*^c^				
Low	*Ref*	–	–	–
Medium	0.283**	–	–	–
	(0.102)			
High	0.453***	–	–	–
	(0.102)			
Married^d^	0.793***	0.766***	0.697***	0.859***
	(0.0587)	(0.246)	(0.0930)	(0.0812)
Age^d^				
18–22	*Ref*	*Ref*	*Ref*	*Ref*
23–27	− 0.278	− 0.376	− 0.152	0.856
	(0.178)	(0.373)	(0.224)	(1.117)
28–32	− 0.215	− 0.563	− 0.105	1.054
	(0.178)	(0.360)	(0.226)	(1.116)
33–39	− 0.796***	− 1.532***	− 0.890***	0.656
	(0.181)	(0.366)	(0.234)	(1.117)
Constant	− 2.109***	− 1.675*	− 1.819***	− 3.041**
	(0.251)	(0.708)	(0.317)	(1.156)
N of person-year observations:	12,000	1173	5029	5734
N of respondents:	8343	825	3449	4069

Data Source: EU-SILC, 24 European countries, 2004–2017

Estimated coefficients with robust standard errors in parentheses; † *p* < 0.10, * *p* < 0.05, ** *p* < 0.01, *** *p* < 0.001; all models include country- and year-fixed effects, and standard errors adjust for clustering within couple in the case of multiple observations of first-birth exposure per couple

^a^First-birth exposure starts in Year t, and continues into Year t + 1 (see Fig. 1)

^b^Full-time, full-year employment status is defined for the 12-month period two years (year_t−2_) before the year t that begins the year of exposure to first birth for both women and their male partners; and additionally three years (year_t−3_) before the year t that begins the year of exposure to first birth for the woman (see again Fig. 1). Year_t−3_ employment status is multiply-imputed when missing due to left-censoring (see text). Magnitudes of differences between estimated probabilities of full-time, full-year employment categories are not statistically different across education levels

^c^Educational attainment categories are: low (pre-primary, primary, lower secondary); medium (upper-secondary, post-secondary non-tertiary); high (tertiary). Education is defined at interview Year t

^d^Age and marital status are defined at interview Year t

**Fig. 4:**
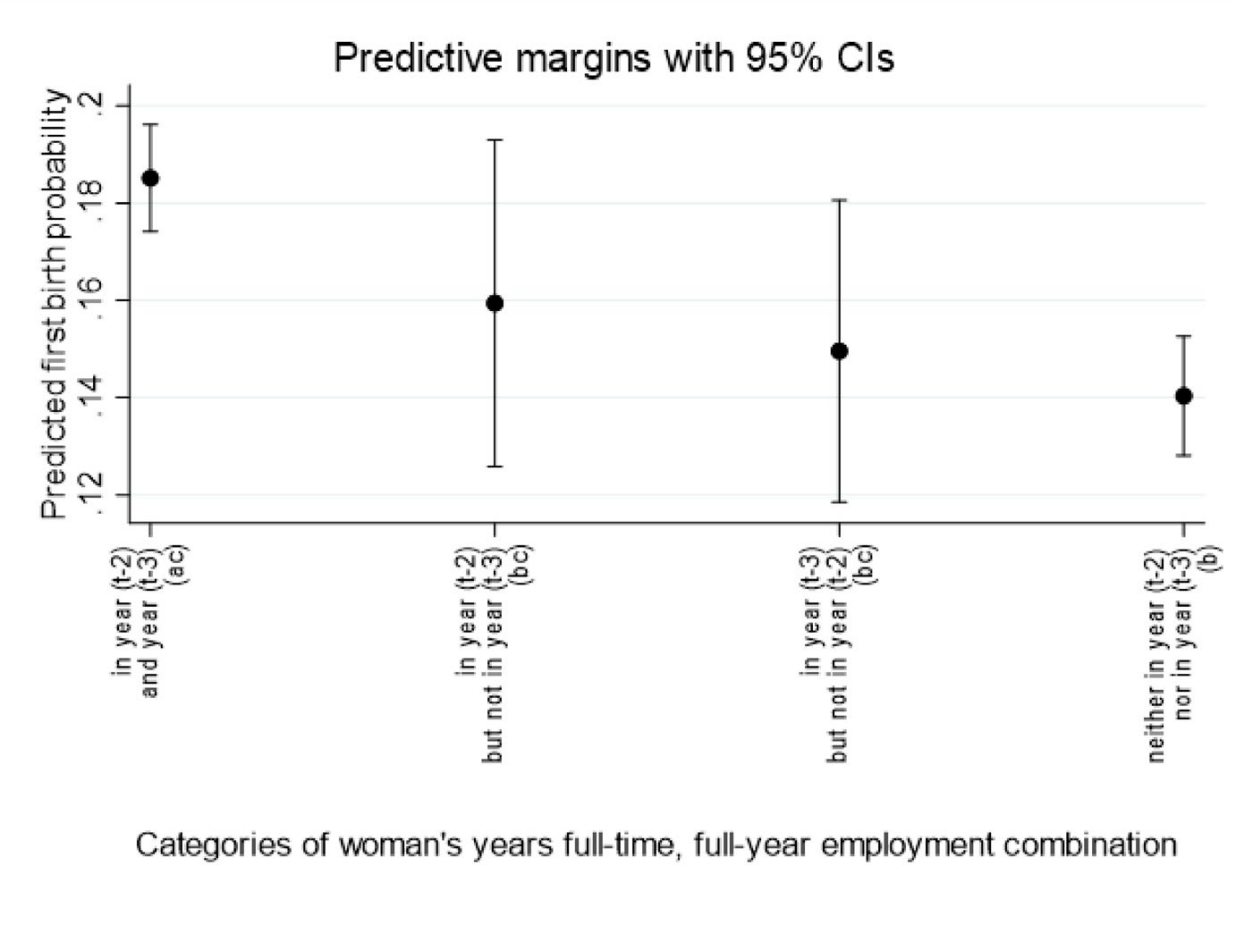
Predicted probability of first birth by woman’s full-time, full-year employment status two and three years before the year of beginning exposure to a first birth*.* Data Source: EU-SILC, 24 European countries, 2004–2017. *Notes:* Predicted first-birth probabilities (at means of covariates) shown are based on regression results presented in Table 3, all women (column 1) with dummy variables to control for educational attainment level. Categories that do not share a script (a, b, c) are statistically different from each other at the 95% confidence level. First-birth exposure starts in Year t, and continues into Year t + 1 (see Fig. 1). Full-time, full-year employment status is defined for the 12-month period two years (year_t−2_) before the year of beginning exposure to first birth; and additionally three years (year_t−3_) before the year of beginning exposure to first birth for women (see again Fig. 1)

Predicted probabilities, derived from the ‘all’ model that pools women across all education levels, with the predicted first birth probability (margins evaluated at the means of other covariates including education) are shown in Fig. 4. The highest first-birth probability is found for women *employed full-time, full-year in both of the two years* (0.185, first category), and lowest is for women who are *full-time, full-year employed in neither of the two years* (0.140, last category). The first-birth probability is the second-highest for women who had *just entered full-time, full-year employment* (0.159), and slightly lower for women who had *just exited from full-time, full-year employment* (0.150). The latter only was found to be statistically significantly different from the first-birth probability of women ‘*full-time, full-year employed in both years*’. This pattern of the highest first-birth probability for women in *full-time, full-year employment in both years* constitutes important evidence that women’s employment stability is associated positively with the couple’s proceeding to a first birth.

Our results from the three separate regression models for *low-, medium-, and high-educated* women are shown in the right three columns of Table 3. The estimated coefficients indicate that for all three educational-attainment groups, the predicted first-birth propensity was highest for women with two consecutive years of full-time, full-year employment. For all three education groups, the coefficient for *not full-time, full-year employed in either year* was substantially and statistically-significantly negative, relative to the reference category of women who are f*ull-time employed in both years*. It is the most striking for *low-educated* women (coefficient value of negative 0.716). However, tests revealed no statistically-significant differences between education groups in the difference in birth probabilities between ‘full-time employed in both years’ versus ‘in neither year’. In summary, we did not find evidence that only for more educated women is there a positive association of their stable employment with partnered first birth. Because we also controlled for male partner’s employment status, our results imply instead, consistent with our third hypothesis, that woman’s stable pre-birth full-time employment is positively associated with the couple’s first-birth risk across all education levels, independently of the man’s employment status.

## Discussion and Conclusion

In our examination of associations between co-residential different-sex couples’ first-birth and the woman’s and the man’s employment statuses in 24 European countries in the period 2004–2017, we found strong evidence in support of our main “gender” hypothesis that first-birth propensities in couples are positively associated not only with the man’s, but also the woman’s, stable employment. This is consistent with theoretical literature suggesting that the dual-earner couple is increasingly normative in Europe (Knight & Brinton, 2017) and increasingly also economically necessary (Goldstein et al., 2013; Zhou & Kan, 2019). It is likely also that the macroeconomic phenomena of increasing economic uncertainty and increasing men’s unemployment have in part led to the dual-earner family model’s overcoming the dominance of the male-breadwinner gender-role model (Alderotti et al., 2021; Zhou & Kan, 2019) through increasing the overall importance to the family of the woman’s earnings (Van Wijk & Billari, 2024). Associated with a dual-earner norm has been an evolving expectation of a life-course trajectory of ongoing labor-force attachment for European women spanning their pre-childrearing and childrearing years in a marital or cohabiting union (Stier et al., 2018), just as it has evolved in the United States (Goldin, 2006; Olivetti, 2006).

Moreover, we found evidence in our analyses of detailed couple labor-force activity statuses that the woman’s stable employment may be even more important than the man’s. Specifically, we found the first-birth propensity is reduced more by the woman’s inactivity than it is by the man’s unemployment or inactivity. We saw this in a statistically-significant difference between the first-birth probabilities for ‘woman full-time, full-year employed; male partner ever-unemployed or ever-inactive’ versus for ‘male partner full-time, full-year employed; woman ever inactive (never unemployed)’.

In support of our second hypothesis that first-birth propensities are positively associated with the woman’s stable employment across country contexts, we found positive associations of women’s stable employment with the couple’s risk of first-birth across European country groups defined by geographic region. We found that both overall and separately for Southern, Western, and Eastern European country-groups, couple combinations in which the woman was not full-time, full-year employed had lower first-birth risks than couple combinations in which both the woman and the man were full-time, full-year employed. This finding that a positive women’s employment association with first birth is general across European regions suggests that the association is independent of a country’s gender-equality norms and employment-family policy context. This differs from previous findings that have contrasted countries by gender equity and that have suggested that while women in higher gender-equity countries (Andersson et al., 2014; Lundström & Andersson, 2012) are more likely to exhibit a positive association of women’s employment with first birth, low gender-equity countries of Southern Europe (Dantis & Rizzi, 2020; Vignoli et al., 2012) exhibit a negative association of women’s employment with first birth. Additionally, across the varied gender-equity contexts of Western European countries, prior studies have found a mix of negative, zero, or positive associations (González & Jurado-Guerrero, 2006; Pailhé & Solaz, 2012; Schmitt, 2012), and prior studies’ findings in Eastern European countries have also been mixed, including zero (Matysiak & Vignoli, 2013) or predominantly negative (Hsu, 2023) associations of women’s employment with first birth.

Our finding of a positive association of women’s stable employment with partnered first births that holds across country contexts is consistent, however, with studies encompassing more recent periods in Italy (Alderotti, 2022; Scherer & Brini, 2023) and in Western Europe (Di Nallo & Lipps, 2023; Schmitt, 2021; van Wijk et al., 2022; Zhou & Kan, 2019). These more recent studies have led investigators to conclude that gender differences in the positive role of employment for fertility are disappearing (Buh, 2023; Schmitt, 2021). In summarizing changes in findings of European studies over time, Buh (2023) described the finding of positive associations of women’s unemployment and unstable employment with fertility in studies for periods through the 1990s as indicating a ‘substitution effect’, whereas the finding that stable employment for both men and women is associated with higher fertility in studies of recent decades indicates the predominance of an ‘income effect’ that increasingly holds for both genders. Consistent with this, in a study of seven high-income countries including three in Europe, Van Wijk and Billari (2024) found direct evidence of an “income-effect” that became positive for women only in the 2010s.

This change over time in the association of women’s employment with fertility in Europe is likely to be one of the major reasons for our finding of uniformly positive women’s and men’s employment and first-birth associations, given this study’s period of first-births 2004–2017. Especially noteworthy here is that whereas González and Jurado-Guerrero (2006) found that combinations of the woman in an ‘inactive’ employment status while the man is full-time employed were associated with a greater first-birth risk than for dual-earner couples across countries observed through the late 1990s, we instead found in the 2004–2017 period that the combination of the woman in an ‘inactive’ employment status while the man is full-time employed had the lowest first-birth risk among all eight combinations of couple employment and labor-force statuses that we considered. Couples in which both the woman and male partner were full-time, full-year employed notably had the highest first-birth risk.

Hsu’s (2023) different findings than ours using the EU SILC across similar years to those of our study, however, suggest that more than a time-period effect is at work. Notably, Hsu found an overall zero, and for some countries positive, association of women’s pre-birth unemployment with first birth, and also found mixed directions of associations with temporary employment, which the author attributed to differences in across countries’ family-policy contexts. We suggest that our study’s measurement of pre-birth employment status with a sufficiently long duration before first-birth exposure is likely to be a critical difference from both Hsu’s study and that of previous work in which employment status has been observed in the year immediately before the birth exposure, inducing a potential endogeneity problem as identified by Wooden et al. (2023) in their analysis of the effects of temporary employment on fertility. An additional problem, moreover, for cross-national studies is that ‘temporary employment’ contracts have different meanings across country contexts and over time (ILO, 2018; Schmitt, 2021). Kopycka et al. (2023) therefore suggested using longitudinal employment observations to better identify employment stability and employment uncertainty for cross-national analyses.

In support of our third hypothesis that first-birth propensities are increased by women’s stable employment for women across educational levels, we found positive associations of stable employment with partnered first births for low, medium, and high education categories. This finding contrasts with several earlier European studies that have found that only or mainly high-educated women have a positive employment association with first birth, including the studies of Kreyenfeld and Andersson (2014) for Germany and Denmark, and of Barbieri et al. (2015) for Italy and Spain. Both sociological (Kreyenfeld, 2010) and economic (del Bono et al., 2012) theoretical mechanisms have been proposed to explain this being possibly due to higher-educated women’s being more ‘career focused’. Moreover, recent U.S. empirical studies similarly align with these European studies, finding that only for college-educated women were there negative effects on first birth of women’s unemployment (Yu & Sun, 2018; and see also Comolli, 2021).

Studies by Meron and Widmer (2002) for France, and by Miettinen and Jalovaara (2020) for women ages 25 and over in Finland, however, found positive employment associations (negative unemployment associations) with first birth that were as high as or higher for lower-educated women as for higher-educated women. Complementing these micro-data findings, Ayllón (2019), using aggregate data for Europe, found a positive employment-fertility association that was greater among those with lower educational attainment. Our finding of a positive association for all education groups, however, does not rule out that the underlying reasons for this association might differ. For example, for lower-educated women, stable employment might facilitate first childbirth due to increased economic certainty, whereas for the higher-educated women, stable employment may instead be a prerequisite for a future career-path trajectory. Again, we suggest that our having measured pre-birth employment based on full-year employment status data observed sufficiently long before the birth-exposure year may be a critical factor in explaining differences between our study’s findings and those of previous studies.

We may summarize our study’s novel methodological contributions to the analysis of the role of women’s employment stability as being two. First, we measured employment over at least a full year rather than at a specific time point. Second, with our use of multiple imputation for left-censored histories, we were able to define women’s full-time, full-year employment status as a predictor variable for up to three years before first birth despite the relatively short (four-year) EU SILC panels. This enabled us to differentiate sequences in which the woman had either recently gained or recently lost full-time full-year employment from sequences that included two consecutive years of full-time full-year employment before exposure to first-birth conception. Moreover, with employment stability defined as full-time, full-year employment in years observed sufficiently before first-birth exposure, the importance of women’s employment stability for the couple’s first birth was seen to be valid across European country groups.

### Limitations

By using EU-SILC data, we were able to cover a large group of European countries and time periods, to allow a sufficient time delay between observation of employment and of exposure to conception, and to observe employment across a full year or two full years rather than to observe employment status at a given moment in time. Our restriction to couples that are co-residential at least for two consecutive waves allowed us to take into account partner characteristics in terms of employment and education, while minimizing confounding with respect to the processes of fertility and of relationship stability. However, with this restriction and with the additional restriction that the woman is not enrolled in full-time education at the beginning of the observed period of exposure to birth, our study is of women that are somewhat older and more educated than average. Beaujouan (2020) noted women’s mean ages at first birth in Europe at around 29 in the period of our study, whereas the weighted median age of those co-residentially partnered and childless in our sample was 31. Moreover, because the woman’s oldest age was necessarily capped at 39 in our sample (to reliably identify nulliparous women), our analyses are of a more concentrated set of ages than for the entire population at risk of a first birth or of a co-residentially partnered first birth. In a supplementary analysis in which all nulliparous women were considered who were co-residentially partnered and not in education, irrespective of the stability of their observed co-residentially partnered years, and irrespective of their having sufficient successive years of observation of employment status before the year of fertility exposure (see section B in the online supplement), the median age remained 31, as in our analytic sample. However, a lower fraction of this less restricted sample were married (54% versus 58%), and a lower fraction were in the highest education category (39% versus 51%).

The implications of the selectivity of our analytic sample for the generalizability of our results, however, may not be large in the European context. Although not all first births are within co-residential partnerships, being stably partnered remains a precondition across European countries for intentions to begin a family (Sturm et al., 2023). Unpartnered childbearing typically accounts for fewer than 10 percent of all births overall, albeit with higher fractions for first births than for higher-order births (Sobotka & Berghammer, 2021, p. 177). Although we were unable to control for unobservable characteristics of ‘stably-partnered’ couples that may favor obtaining stable employment as a precondition for first couple birth, we did control for the observable characteristics of marital status and educational attainment in our main regression models. Moreover, in the component of our study that stratified the sample into low, medium, and high education groups, we found that the association between stable employment and first birth was not statistically different across the three education groups when we incorporated up to two prior years of full-time, full-year employment status.

Our excluding those who were in full-time schooling two years and three years before the first-birth exposure interval in our study is also unlikely to be a major problem for the generalizability of our findings. Family formation immediately after completing schooling remains relatively rare, and instead a lengthening of participation in full-time schooling and consequent older ages of education completion has been a primary factor responsible for increases in ages at first birth across European countries (Neels et al., 2017; Vasireddy et al., 2023). Nevertheless, women with low educational attainment may have been selectively omitted from our analyses to the extent that their conception or birth occurred while still in, or very soon after leaving, full-time education. This could lead to an overstatement of the extent to which stable full-time, full-year employment is associated with higher first-birth propensities for low-educated women.

A major strength of our study is that we included both cohabitation and marriage as contexts for first co-residentially partnered childbearing, as many first births in Europe are within non-marital cohabiting unions, though with substantial variation across countries (Sobotka & Berghammer, 2021). We included being married only as a control variable in our analyses. However, as getting married itself may be hastened by a conception (Holland, 2017), this would lead to a spuriously high married versus cohabiting coefficient if interpreted as a causal effect of being married. We conducted sensitivity analyses in which we omitted marital status from our regression models, and found that the estimates of the coefficient for our main, employment-status predictor variables were substantially unchanged (results presented in Table S2, columns 2 and 4, in the online supplement).

### Policy Implications

In summary, by re-evaluating the association between women’s employment and partnered first births in Europe, our study was able to generate a coherent and consistent picture of the importance of employment stability of both women and men across European country contexts and educational attainment levels. We conclude that the era of the male-breadwinner couple is increasingly a phenomenon of the past as couples across Europe prepare for their family formation. Our study also demonstrates that it will be crucial in future research to adequately capture employment stability and to assess this sufficiently earlier than the period of fertility exposure, when assessing the micro-level associations between fertility and economic context. Exploring different employment-stability operationalizations and concepts in a comparative setting also seems to us a very fruitful approach.

Low fertility in Europe and other high-income countries continues to be a major policy concern (Bloom et al., 2024). Our finding of a positive association between women’s stable employment and first birth that exists across European countries does not undermine the importance of socio-economic and family policies in shaping fertility reactions to uncertain employment conditions of young adults in Europe. They rather suggest that for starting a family, couples across European countries and education groups are, besides family policies encouraging gender-neutral work/life balance options (formal childcare, remunerated parental leave), increasingly in need of economic policies ensuring stable, long-term employment. In many European countries, a dual-earner norm co-exists with greater challenges for the woman than for the man in the couple to continue their employment trajectory once childbearing begins (Ayllón, 2019; Bellido & Marcén, 2019). Our study provides new evidence indicating that the woman’s employment stability has become a critical precondition for the couple’s beginning childbearing across Europe. Moreover, our evidence suggests not only that women’s stable employment is now no less important than men’s, but that it may be even more important.

## Supplementary Information

Below is the link to the electronic supplementary material.

Supplementary Material 1

## Footnotes

1 France and Portugal follow households for more than four annual waves, but we only used the first four waves to obtain a harmonized panel data set in which each country is subject to the same period of exposure.

2 We used a script provided by Borst (2018) to merge these data sets into one cumulative dataset (containing years 2004 to 2017).

3 Austria, Belgium, Cyprus, Czech Republic, Denmark, Estonia, Finland, France, Greece, Hungary, Ireland, Italy, Lithuania, Latvia, Malta, Netherlands, Poland, Portugal, Serbia, Spain, Switzerland, Sweden, Slovenia, United Kingdom.

4 Greulich and Dasré (2017) found that from age 40 on, EU-SILC-measures of period fertility by parity are potentially biased as older children might already have left the parental household.

5 This employment status was observed on a monthly basis, reported for the preceding calendar year (codes as PL210A-F, respectively PL211A-F in EU-SILC, depending on the waves). Eurostat reported a very small percentage of missingness. For example, for the latest longitudinal wave used in our study, Eurostat reported an overall missingness of 6.2%. Of this, 5.1% was due to ‘not selected respondent’ (information given by the ‘flag’-variable that provided supplementary information to the value of the main variable). This left 1.1% of unexplained missingness (GESIS—Leibniz-Institute for the Social Sciences, 2022). Statistics on missingness for this variable were similar for earlier waves.

6 Our regression results were robust to controls for time between observed labor force status and beginning of exposure to birth, for variation in months of exposure to birth, and for number of observed waves per individual (three versus four). We excluded these technical components from the regression specifications presented in the paper’s main findings after finding that none were statistically significant nor had a substantive effect on the regression estimates.

7 Another motivation for our not including both labor-market status and income was to avoid excessive multicollinearity: Spearman’s rho correlation coefficients were relatively high, for example 0.59 between female labor market status and female labor income, respectively 0.41 between female labor market status and disposable household labor income (employee cash or near cash income as well as cash benefits or losses from self-employment of both the woman and her partner, observed for the same calendar year for which we observed the labor market status). Pearson’s Chi-squared and Fisher’s exact tests indicated that the hypothesis of independence must be rejected (p < 0.001).

8 We did not estimate our model separately for the countries in a ‘Northern Europe’ region due to its small sample size (around 900 person-years, see Notes to Table 2).

9 For country-group interactions between region and couple employment, we used Stata’s *margins, dydx* command. We tested contrasts of average marginal effects with the *margins, dydx contrast (atcontreast(i) pveffects predict(pr)* command. For interactions term between our three-category variable of educational attainment and our four-category variable of woman’s employment stability, with the regression model estimated using multiple imputation for left-censored employment status at *t-3*, we used Stata’s *mimrgns, dydx* command to test for statistical significance of average marginal effects. We tested contrasts of average marginal effects with the *mimrgns, dydx contrast (atcontreast(i) pveffects predict(pr)* command.

10 Marital status may be endogenous, with couples deciding to marry because they intend to have a child. Because we observed marital status at the interview wave marking the beginning of the fertility-exposure year, this may partly explain the large magnitude of the ‘married’ coefficient across our models. In sensitivity analyses, we alternately excluded the marital status variable and found that our main results (those for the employment-status coefficients) were not affected.

11 Although the estimated first-birth probability values were somewhat higher for couples with *only the woman FT employed* compared to couples with *only the male partner FT employed*, the difference between these two probabilities was not statistically significant.

12 In results not reported here, we also found a large and negative (-0.520) coefficient for ‘neither *full-time, full-year employed*’ for Northern European countries, but that was not statistically significantly different from zero. The Northern Europe group unfortunately suffered from low statistical power due to its small sample size (see Notes to Table 2).

13 Statistical tests were based on the coefficients of the model of the ‘all’ column, successively changing the reference category of the employment variable. Results (not shown) indicated that the difference between women ‘employed in year *t-2*, but not in year *t-3’* and each of the other three women’s employment categories were not statistically significant.

14 We obtained this information by successively changing the education reference category in Table 3’s column 1.

## References

[CR1] Adsera, A. (2011). Where are the babies? Labor market conditions and fertility in Europe. *European Journal of Population,**27*(1), 1–32. 10.1007/s10680-010-9222-x 23580794 10.1007/s10680-010-9222-x PMC3620442

[CR2] Ahn, N., & Mira, P. (2002). A note on the changing relationship between fertility and female employment rates in developed countries. *Journal of Population Economics,**15*(4), 667–682. 10.1007/s00148-003-0166-x

[CR3] Alderotti, G. (2022). Female employment and first childbirth in Italy: What news? *Genus,**78*, 14. 10.1186/s41118-022-00162-w

[CR87] Alderotti, G., Mussino, E., &, and Comolli, C.L. (2023). Natives’ and migrants’ employment uncertainty and childbearing during the great recession: a comparison between Italy and Sweden. *European Societies,**25*(4), 539–573. 10.1080/14616696.2022.2153302

[CR4] Alderotti, G., Vignoli, D., Baccini, M., & Matysiak, A. (2021). Employment instability and fertility in Europe: A meta-analysis. *Demography,**58*(3), 871–900. 10.1215/00703370-9164737 33899914 10.1215/00703370-9164737

[CR5] Andersen, S. H., & Özcan, B. (2021). The effects of unemployment on fertility. *Advances in Life Course Research*. 10.1016/j.alcr.2020.100401 10.1016/j.alcr.2020.100401 36695115

[CR6] Andersson, G., Kreyenfeld, M., & Mika, T. (2014). Welfare state context, female labour-market attachment and childbearing in Germany and Denmark. *Journal of Population Research,**31*, 287–316. 10.1007/s12546-014-9135-3

[CR7] Arpino, B., Esping-Andersen, G., & Pessin, L. (2015). How do changes in gender role attitudes towards female employment influence fertility? A macro-level analysis. *European Sociological Review,**31*(3), 370–382. 10.1093/esr/jcv002

[CR8] Ayllón, S. (2019). Job insecurity and fertility in Europe. *Review of Economics of the Household,**17*, 1321–1347. 10.1007/s11150-019-09450-5

[CR9] Barbieri, P., Bozzon, R., Scherer, S., Grotti, R., & Lugo, M. (2015). The rise of a Latin model? Family and fertility consequences of employment instability in Italy and Spain. *European Societies,**17*(4), 423–444. 10.1080/14616696.2015.1064147

[CR10] Beaujouan, E. (2020). Latest-late fertility? Decline and resurgence of late parenthood across the low-fertility countries. *Population and Development Review,**46*(2), 219–247. 10.1111/padr.12334 32733116 10.1111/padr.12334 PMC7384131

[CR11] Becker, G. S. (1960). *An economic analysis of fertility*. Princeton University Press.

[CR12] Bellido, H., & Marcén, M. (2019). Fertility and the business cycle: The European case. *Review of Economics of the Household,**17*, 1289–1319. 10.1007/s11150-019-09449-y

[CR13] Bloom, D. E., Kuhn, M., & Prettner, K. (2024). Fertility in high-income countries: Trends, patterns, determinants, and consequences. *Annual Review of Economics,**16*, 159–184. 10.1146/annurev-economics-081523-013750

[CR14] Borst, M. (2018). *EU-SILC Tools: EU-SILC panel. First computational steps towards a cumulative sample based on the EU-SILC longitudinal datasets.* GESIS Papers 2018/11, Mannheim. 10.21241/ssoar.57347

[CR15] Brinton, M. C., & Lee, D.-J. (2016). Gender-role ideology, labor market institutions, and post-industrial fertility. *Population and Development Review,**42*(3), 405–433. 10.1111/padr.161

[CR16] Buh, B. (2023). Measuring the effect of employment uncertainty on fertility in low-fertility contexts: An overview of existing measures. *Genus,**79*, 4. 10.1186/s41118-023-00185-x 36760753 10.1186/s41118-023-00185-x PMC9904270

[CR17] Comolli, C. L. (2021). Couples’ paid work, state-level unemployment, and first births in the United States. *Demographic Research,**45*, 1149–1184. 10.4054/DemRes.2021.45.38

[CR18] Cotter, D. A., Hermsen, J. M., & Vanneman, R. (2011). The end of the gender revolution? Gender role attitudes from 1977 to 2008. *American Journal of Sociology,**117*(1), 259–289. 10.1086/658853 10.1086/658853 22003521

[CR19] D’Albis, H., Greulich, A., & Ponthière, G. (2017). Education, labour, and the demographic consequences of birth postponement in Europe. *Demographic Research,**36*, 691–728. 10.4054/DemRes.2017.36.23

[CR20] Dantis, C., & Rizzi, E. L. (2020). Transition to first birth during the Great Recession: The case of Greece. *Genus*. 10.1186/s41118-019-0070-1

[CR21] Del Bono, E., Weber, A., & Winter‐Ebmer, R. (2012). Clash of career and family: On couples’ fertility decisions after job displacement. *Journal of the European Economic Association,**10*(4), 659–683. 10.1111/j.1542-4774.2012.01074.x

[CR22] Di Nallo, A., & Lipps, O. (2023). How much his or her job loss influences fertility: A couple approach. *Journal of Marriage and Family,**85*, 873–897. 10.1111/jomf.12907

[CR23] Doepke, M., Hannusch, A., Kindermann, F., & Tertilt, M. (2023). The economics of fertility: A new era. In S. Lundberg & A. Voena (Eds.), *Handbook of the economics of the family* (Vol. 1, pp. 151–254). Elsevier. 10.1016/bs.hefam.2023.01.003

[CR24] Engelhardt, H., Kögel, T., & Prskawetz, A. (2004). Fertility and women’s employment reconsidered: A macro-level time-series analysis for developed countries. *Population Studies,**58*(1), 109–120. 10.1080/0032472032000167715 15204266 10.1080/0032472032000167715

[CR25] England, P. (2010). The gender revolution: Uneven and stalled. *Gender & Society,**24*(2), 149–166. 10.1177/0891243210361475

[CR26] Esping-Andersen, G., & Billari, F. C. (2015). Re-theorizing family demographics. *Population and Development Review,**41*(1), 1–31. 10.1111/j.1728-4457.2015.00024.x

[CR27] Eurostat. (2012). *Comparative EU final quality report.* Version 3, July 2011 [technical report] https://doc.ukdataservice.ac.uk/doc/6729/mrdoc/pdf/6729_eurostat_prodcom_quality_report_2012.pdf

[CR28] Eurostat. (2023). *EU-SILC microdata, 2004–2017 (Version 1).* https://ec.europa.eu/eurostat/web/microdata/european-union-statistics-on-income-and-living-conditions

[CR29] GESIS – Leibniz Institute for the Social Sciences. (2022). *Codebook EU-SILC 2017 panel file.* [technical report] https://www.gesis.org/missy/files/documents/EU-SILC/Codebook_EU-SILC-2017-panel.pdf

[CR30] Goldin, C. (2006). The quiet revolution that transformed women’s employment, education, and family. *American Economic Review,**96*(2), 1–21. 10.1257/000282806777212350

[CR31] Goldscheider, F., Bernhardt, E., & Lappegård, T. (2015). The gender revolution: A framework for understanding family and demographic behavior. *Population and Development Review,**41*(2), 207–239. 10.1111/j.1728-4457.2015.00045.x

[CR32] Goldstein, J., Kreyenfeld, M., Jasilioniene, A., & Örsal, D. (2013). Fertility reactions to the ‘Great Recession’ in Europe: Recent evidence from order-specific data. *Demographic Research,**29*(4), 85–104. 10.4054/DemRes.2013.29.4

[CR85] Gornick, J.C., Meyers, M.K., & Ross, K.E. (1997). Supporting the employment of mothers: Policy variation across fourteen welfare states. *Journal of European Social Policy,**7*(1), 45–70. 10.1177/095892879700700103

[CR33] González, M. J., & Jurado-Guerrero, T. (2006). Remaining childless in affluent economies: A comparison of France, West Germany, Italy and Spain, 1994–2001. *European Journal of Population,**22*(4), 317–352. 10.1007/s10680-006-9000-y

[CR34] Greulich, A., & Dasré, A. (2017). Quality of periodic fertility measures in EU-SILC. *Demographic Research,**36*, 525–556. 10.4054/DemRes.2017.36.17

[CR35] Hofmann, B., Kreyenfeld, M., & Uhlendorff, A. (2017). Job displacement and first birth over the business cycle. *Demography,**54*, 933–959. 10.1007/s13524-017-0580-4 28585024 10.1007/s13524-017-0580-4 PMC5486876

[CR36] Holland, J. A. (2017). The timing of marriage vis-à-vis coresidence and childbearing in Europe and the United States. *Demographic Research,**36*, 609–626. 10.4054/DemRes.2017.36.20

[CR37] Hook, J. L., & Paek, E. (2020). A stalled revolution? Change in women’s labor force participation during child-rearing years, Europe and the United States 1996–2016. *Population and Development Review,**46*(4), 677–708. 10.1111/padr.12364

[CR38] Hsu, C.-H. (2023). How women’s employment instability affects birth transitions: The moderating role of family policies in 27 European countries. *European Sociological Review,**39*(6), 935–956. 10.1093/esr/jcad037

[CR39] Huttunen, K., & Kellokumpu, J. (2016). The effect of job displacement on couples’ fertility decisions. *Journal of Labor Economics,**34*(2), 403–442. 10.1086/68364

[CR40] Iacovou, M., Kaminska, O., & Levy, H. (2012). *Using EU-SILC data for cross-national analysis: Strengths, problems and recommendations.* ISER Working Paper Series, No. 2012-03, Institute for Social and Economic Research. https://www.econstor.eu/bitstream/10419/65951/1/686613252.pdf

[CR41] ILO. (2018). *Working time and the future of work,* Geneva*.* https://www.ilo.org/publications/working-time-and-future-work

[CR42] Kalmijn, M. (2013). The educational gradient of marriage: A comparison of European countries. *Demography,**50*, 1499–1520. 10.1007/s13524-013-0229-x 23821472 10.1007/s13524-013-0229-x

[CR43] Knight, C. R., & Brinton, M. C. (2017). One egalitarianism or several? Two decades of gender role attitude change in Europe. *American Journal of Sociology,**122*(5), 1485–1532. 10.1086/689814

[CR44] Kopycka, K., Kiersztyn, A., Sawinski, Z., Bienkowski, S., & Sovpenchuk, V. (2023). Use of panel surveys to measure employment precarity in a cross-national framework: An integrated approach to harmonize research concepts and longitudinal data. *Survey Research Methods,**17*(3), 353–393. 10.18148/srm/2023.v17i3.7989

[CR45] Kravdal, Ø. (2002). The impact of individual and aggregate unemployment on fertility in Norway. *Demographic Research,**6*(10), 263–294. 10.4054/DemRes.2002.6.10

[CR46] Kreyenfeld, M. (2010). Uncertainties in female employment careers and the postponement of parenthood in Germany. *European Sociological Review,**26*, 351–366. 10.1093/esr/jcp026

[CR47] Kreyenfeld, M., & Andersson, G. (2014). Socioeconomic differences in the unemployment–fertility nexus: Evidence from Denmark and Germany. *Advances in Life Course Research,**21*, 59–73. 10.1016/j.alcr.2014.01.007 26047542 10.1016/j.alcr.2014.01.007

[CR48] Kristensen, A., & Lappegård, T. (2022). Unemployment and fertility: The relationship between individual and aggregated unemployment and fertility during 1994–2014 in Norway. *Demographic Research*. 10.4054/DemRes.2022.46.35

[CR49] Kunze, A. (2018). The gender wage gap in developed countries. In S. L. Averett, L. M. Argys, & S. D. Hoffman (Eds.), *Oxford handbook of women and the economy. *Oxford University Press.

[CR50] Lass, I. (2020). The effects of non-standard employment on the transition to parenthood within couples: A comparison of Germany and Australia. *European Journal of Population,**36*, 843–874. 10.1007/s10680-019-09548-7 33184560 10.1007/s10680-019-09548-7 PMC7642039

[CR51] Lesthaeghe, R. (2010). The unfolding story of the second demographic transition. *Population and Development Review,**36*(2), 211–251. 10.1111/j.1728-4457.2010.00328.x 20734551 10.1111/j.1728-4457.2010.00328.x

[CR52] Lundström, K. E., & Andersson, G. (2012). Labor market status, migrant status and first childbearing in Sweden. *Demographic Research,**27*, 719–742. 10.4054/DemRes.2012.27.25

[CR53] Matysiak, A., & Vignoli, D. (2013). Diverse effects of women’s employment on fertility: Insights from Italy and Poland. *European Journal of Population,**29*, 273–302. 10.1007/s10680-013-9287-4 23956480 10.1007/s10680-013-9287-4 PMC3744382

[CR54] McDonald, P. (2000). Gender equity, social institutions and the future of fertility. *Journal of Population Research,**17*(1), 1–16. 10.1007/BF03029445

[CR55] McDonald, P. (2006). Low fertility and the state: The efficacy of policy. *Population and Development Review,**32*(3), 485–510. 10.1111/j.1728-4457.2006.00134.x

[CR56] Meron, M., & Widmer, I. (2002). Unemployment leads women to postpone the birth of their first child. *Population,**57*(2), 301–330. 10.2307/3246611

[CR57] Miettinen, A., & Jalovaara, M. (2020). Unemployment delays first birth but not for all. Life stage and educational differences in the effects of employment uncertainty on first births. *Advances in Life Course Research,**43*, 100320. 10.1016/j.alcr.2019.100320 36726257 10.1016/j.alcr.2019.100320

[CR58] Musick, K., Doherty Bea, M., & Gonalons-Pons, P. (2020). His and her earnings following parenthood in the United States, Germany, and the United Kingdom. *American Sociological Review,**85*(4), 639–674. 10.1177/0003122420934430

[CR59] Neels, K., Murphy, M., Ní Bhrolcháin, M., & Beaujouan, E. (2017). Rising educational participation and the trend to later childbearing. *Population and Development Review,**43*(4), 667–693. 10.1111/padr.12112 29398739 10.1111/padr.12112 PMC5767733

[CR60] Neyer, G., Lappegård, T., & Vignoli, D. (2013). Gender equality and fertility. Which equality matters? *European Journal of Population,**29*, 245–272. 10.1007/s10680-013-9292-7

[CR61] Olivetti, C. (2006). Changes in women’s hours of market work: The role of returns to experience. *Review of Economic Dynamics,**9*, 557–587. 10.1016/j.red.2006.06.001

[CR62] Oppenheimer, V. K. (1988). A theory of marriage timing. *American Journal of Sociology,**94*(3), 563–591. 10.1086/229030

[CR63] Oshio, T. (2019). Is a positive association between female employment and fertility still spurious? *Demographic Research,**41*, 1277–1288. 10.4054/DemRes.2019.41.45

[CR64] Pailhé, A., & Solaz, A. (2012). The influence of employment uncertainty on childbearing in France: A tempo or quantum effect? *Demographic Research,**26*, 1–40. 10.4054/DemRes.2012.26.1

[CR66] Rendall, M. S., & Greulich, A. (2016). Multiple imputation for demographic hazard models with left-censored predictor variables: Application to employment duration and fertility in the EU-SILC. *Demographic Research,**35*, 1135–1148. 10.4054/DemRes.2016.35.38 33273886 10.4054/demres.2016.35.38 PMC7710159

[CR67] Rindfuss, R. R. (1976). Annual fertility rates from census data on own children: Comparisons with vital statistics data for the United States. *Demography,**13*(2), 235–249. 10.2307/2060803 1278582

[CR68] Schaller, J. (2016). Booms, busts, and fertility: Testing the Becker model using gender-specific labor demand. *Journal of Human Resources,**51*(1), 1–29. 10.3368/jhr.51.1.1

[CR69] Scherer, S., & Brini, E. (2023). Employment instability and childbirth over the last 20 years in Italy. *European Journal of Population,**39*, 31. 10.1007/s10680-023-09680-5 37823967 10.1007/s10680-023-09680-5 PMC10570255

[CR70] Schmitt, C. (2012). A cross-national perspective on unemployment and first births. *European Journal of Population,**28*, 303–335. 10.1007/s10680-012-9262-5

[CR71] Schmitt, C. (2021). The impact of economic uncertainty, precarious employment, and risk attitudes on the transition to parenthood. *Advances in Life Course Research*. 10.1016/j.alcr.2021.100402 10.1016/j.alcr.2021.100402 36695145

[CR72] Sobotka, T., & Berghammer, C. (2021). Demography of family change in Europe. In Schneider & Kreyenfeld (Eds.), *Handbook on the sociology of the family. *Edward Elgar. 10.4337/9781788975544.00019

[CR73] Stier, H., Lewin-Epstein, N., & Braun, M. (2018). Institutional change and women’s work patterns along the family life course. *Research in Social Stratification and Mobility,**57*, 46–55. 10.1016/j.rssm.2018.07.001

[CR74] Sturm, N., Koops, J. C., & Rutigliano, R. (2023). The influence of partnership status on fertility intentions of childless women and men across European countries. *European Journal of Population,**39*, Article 20. 10.1007/s10680-023-09664-5 37395831 10.1007/s10680-023-09664-5 PMC10317918

[CR86] Thévenon, O. (2015). Institutional settings of having children: A comparison of family policy development across Europe. In J. Klobas, A. Liefbroer, & D. Philipov (Eds.), Reproductive Decision Making in a Micro-Macro Context. Springer.

[CR75] Sweeney, M. M. (2002). Two decades of family change: The shifting economic foundations of marriage. *American Sociological Review,**67*(1), 132–147. 10.2307/3088937

[CR76] Van Wijk, D. C., de Valk, H. A., & Liefbroer, A. C. (2022). Economic precariousness and the transition to parenthood: A dynamic and multidimensional approach. *European Journal of Population,**38*, 457–483. 10.1007/s10680-022-09617-4 35966358 10.1007/s10680-022-09617-4 PMC9363546

[CR77] Van Wijk, D., & Billari, F. C. (2024). Fertility postponement, economic uncertainty, and increasing income prerequisites of parenthood. *Population and Development Review,**50*(2), 287–322. 10.1111/padr.12624

[CR78] Vasireddy, S., Berrington, A., Kuang, B., & Kulu, H. (2023). Education and fertility: A review of recent research in Europe. *Comparative Population Studies,**48*, 553–588. 10.12765/CPoS-2023-21

[CR79] Vignoli, D., Drefahl, S., & De Santis, G. (2012). Whose job instability affects the likelihood of becoming a parent in Italy? A tale of two partners. *Demographic Research,**26*, 41–62. 10.4054/DemRes.2012.26.2

[CR80] Wielers, R., & Raven, D. (2013). Part-time work and work norms in the Netherlands. *European Sociological Review*. 10.1093/esr/jcr043

[CR81] Wooden, M., Trinh, T.-A., & Mooi-Reci, I. (2023). The differential impacts of contingent employment on fertility: Evidence from Australia. *Social Forces,**102*(1), 330–352. 10.1093/sf/soad008

[CR82] Yu, W.-h, & Sun, S. (2018). Fertility responses to individual and contextual unemployment: Differences by socioeconomic background. *Demographic Research,**39*, 927–962. 10.4054/DemRes.2018.39.35

[CR83] Zaidi, B., & Morgan, S. P. (2017). The second demographic transition theory: A review and appraisal. *Annual Review of Sociology,**43*(1), 473–492. 10.1146/annurev-soc-060116-053442 10.1146/annurev-soc-060116-053442 PMC5548437 28798523

[CR84] Zhou, M., & Kan, M.-Y. (2019). A new family equilibrium? Changing dynamics between the gender division of labor and fertility in Great Britain, 1991–2017. *Demographic Research,**40*, 1455–1500. 10.4054/DemRes.2019.40.50

