# Supplementary material for: An Ongoing Gender Revolution in Europe: Women’s Stable Employment as a Precondition for Partnered First Births
Source: Popul Res Policy Rev. 2026 Feb 9;45(1):8. doi: 10.1007/s11113-026-09990-6 (PMC12886254; doi:10.1007/s11113-026-09990-6)
Supplement: Supplementary file 1 — Supplementary Material 1 [file 11113_2026_9990_MOESM1_ESM.docx]

**ONLINE SUPPLEMENT**

**An Ongoing Gender Revolution in Europe:**

**Women’s Stable Employment as a Precondition for Partnered First Births.**

***A. Country Exclusions in the Sample***

Germany was not covered in the longitudinal module of EU-SILC, because Germany does not release its panel data due to data confidentiality reasons and because of non-conforming protocols with respect to probability sampling (Iacovou et al., 2012). We dropped Norway, Iceland, and Luxembourg due to coding-harmonization problems in the longitudinal module. Persistent coding-harmonization issues that complicate the integration of country-specific data into cumulative, multi-year datasets can arise due to national data collection autonomy which can lead to inconsistencies in variable coding, especially in countries with unique administrative or statistical systems like Norway, Iceland, and Luxembourg (Borst, 2018). France and Portugal follow households for more than four annual waves, but we only used the first four waves to obtain a harmonized panel data set in which each country was subject to the same period of exposure. We also dropped Romania, Bulgaria, Croatia and Slovakia due to fertility measurement deficiencies, as explained in more detail in the next section of this Online Supplement.

***B. Selection Bias***

To focus on the employment-fertility nexus, we excluded women who were students or in further training at the beginning of exposure to first birth. Furthermore, our analytic sample contains only women who were observed for at least three waves and who were continuously partnered for at least two waves. This extended time period allowed us to measure employment stability within the couple over a relatively long time period (12 months) as well as to reduce potential endogeneity of employment with conception and birth. However, these sample restrictions reduced the generalizability of our study to some extent. In a supplementary analysis, we compared the characteristics of our analytical sample to others without restrictions (see Supplementary Table S1).

[Table S1 about here]

Our analytical sample consists of the 12,060 women who were not students or in further training at the beginning of exposure to first birth and who were coresidentially partnered in both years t and t+1, being the beginning and end-point of the fertility-exposure year (first column of table A). As we showed also in our study’s descriptive-statistics Table 1, this sample’s sociodemographic characteristics that were observed at year t include that 50.8% had ‘high’ (tertiary) educational attainment, 58.1% were married, and their median age was 31. If we remove the restriction that they are coresidentially partnered in both years t and t+1 and consider instead all those who were coresidentially partnered at the previous wave, at year t-1, our sample size would be 20,510 (second column of Table S1). Of these, 39.2% had ‘high’ (tertiary) educational attainment, 53.9% were married, and their median age was again 31. To add to this comparison, we also considered their employment status as observed at the time of the survey. Here, we see that 68.5% were full-time employed at time t for the 12,060-person analytical sample of our study, whereas 60.4% were full-time employed at time t-1 for the larger, 20,510-person sample. This supplementary comparison therefore suggests that our analytical sample is somewhat positively selected on socioeconomic characteristics due to the focus on women who were continuously partnered *for at least two waves* rather than partnered at the previous wave only. The generalizability of our results is therefore to this positively-selected stably-coresident group. We controlled for age throughout our analyses, and we either controlled for, or stratified by, education in the main regression analyses of Tables 2 and 3.

The additional exclusion of women who were not students or in further training at the beginning of exposure to first birth further does, however, not increase selectivity: Compared to column two, the third column of Table S1 shows very similar socioeconomic characteristics for the sample of women who were coresidentially partnered at the previous wave, at year t-1, including those who were still in education at the beginning of exposure to first birth. Finally, column four shows descriptive statistics of the overall sample of childless women aged 18 to 39, independent of their partner status and including those who were still in education. Women in this much larger sample (N=116,026) were naturally younger (their median age is 27) and lower educated (28.1% have high educational attainment).

***C. Different Ways of Measuring Employment Stability***

Labor-force status observed only at a given point in time (e.g., at the time of the survey interview) is in general a weaker indicator of the continuity and stability of employment. The EU-SILC provides information about the type of employment contract (fixed term versus permanent). However, this kind of information does not provide an indicator of employment security that is consistent across all European countries, as having a temporary job does not provide the same level of precariousness across countries (ILO, 2018). Also, information about ‘contract type’ in the EU-SILC is only available for employment observed at the time of the survey. Our employment measure instead not only includes monthly information for a full year, but also for the calendar year prior to the first survey-interview year for each panel. Our indicator of employment stability as full-time, full-year employment may still capture employment security differently across societies (depending on risk of unemployment, for example), but cross-national variation in the meaning of this measure is likely to be much less than that for ‘contract type’ measures at a single point in time (Kopycka et al., 2023). Additionally, we were able to be much more confident about overcoming the endogeneity problem seen with ‘contract type’ measured close to the period of birth exposure (Wooden et al 2023).

***D. Potential Biases in the Measurement of Fertility***

The “own-children” method is a common technique for reconstructing women's fertility behavior in the absence of retrospective birth histories. It is often used with data that includes demographic information of all household members, but no retrospective fertility histories (Desplanques, 1994). Two potential biases in the measurement of fertility arise, however, with the “own-children” method: First, there may be unobserved children living outside the household: fertility levels could thereby be underestimated and birth orders not correctly attributed. Second, longitudinal data may be affected by fertility-linked attrition, as childbirth can cause the individual or household to move, leading to an underrepresentation of households with young children. The sampling and the weighting procedures in EU-SILC are not directly designed to ensure unbiased fertility estimation. Greulich and Dasré (2017) quantified the measurement bias in period fertility in EU-SILC and found a systematic fertility underestimation. However, for the majority of covered countries, the underestimation did not exceed 10%. We excluded from the present study the four countries (Romania, Bulgaria, Croatia and Slovakia) with higher rates of underestimation than this. In addition, and most importantly, Greulich and Dasré (2017) found no socioeconomic differentials in attrition in the longitudinal sample. This suggests that our analysis of socio-economic differentials in the transition to first births is unlikely to be affected by fertility-linked attrition and/or sample selection.

***E. Multiple imputation***

Multiple imputation (MI) for left-censored data was used to retain the same sample sizes for our *t-3* and *t-2* employment-status models as for our *t-2*-only employment-status models. We followed the methodology developed and implemented for one EU SILC country (Poland) in Rendall and Greulich (2016). For the ‘incomplete’ cases in which observation sequences are of only three waves, the woman’s prior year’s (*t-3*) employment was imputed from the ‘complete’ cases of observed sequences of four waves. All standard errors for those models used standard algorithms for incorporating imputation uncertainty. Our observation plan, consisting of three consecutive years of panel observation for each first-birth exposure, provided a 24-country 2004-2017 EU-SILC dataset of 12,060 person-years of first-birth exposure. These person-years were contributed by 8,386 women: 56% contributed one person-year of first-birth exposure (women observed for three consecutive waves) and 44% contributed two person-years of first-birth exposure (women observed for four consecutive waves). Of these two person-years, one included two years of employment-status observation and the other year had one observed year and one left-censored year of employment-status observation. This constituted a ‘missing-by-design’ (Raghunathan and Grizzle, 1995) implementation of MI. It has the major advantage over MI for non-response missingness that random sampling into the survey, in our case into a given EU-SILC panel, is the process that generates the missingness (the left-censored employment-status year). Therefore, the Missing At Random (MAR) assumption required for unbiased MI was straightforwardly satisfied. Crucially, by using MI for left-censored employment histories, we were able to estimate a model that includes year *t-2* and *t-3* employment-status predictor variables with no reduction in sample size over that in which at least year *t-2* (but not necessarily year *t-3*) was observed. In particular, while retaining the full 12,060 person-years of first-birth exposure, we were able to specify a model which included as predictor categories whether the woman is *full-time, full-year employed in both of the two years t-3 and t-2*, whether she was *not* *full-time, full-year employed in either of the two years t-3 and t-2*, whether she was *full-time, full-year employed only in the earlier of the two years t-3* ---- that is, she has recently exited full-time, full-year employment ---- and whether she was *full-time, full-year employed only in the later of the two years t-2* ---- that is, she has recently entered full-time, full-year employment.

**References:**

Borst, M. (2018). *EU-SILC Tools: EU-SILC panel.* *First computational steps towards a cumulative sample based on the EU-SILC longitudinal datasets.* GESIS Papers 2018/11, Mannheim. <https://doi.org/10.21241/ssoar.57347>

Desplanques G. (1994): Measuring fertility differentials from census information alone. *Population: An English Selection* 6: 23-33. <https://www.jstor.org/stable/2949142?seq=1>

Greulich, A., & Dasré, A. (2017). Quality of periodic fertility measures in EU-SILC. *Demographic Research,* 36(17), 525–556. <https://doi.org/10.4054/DemRes.2017.36.17>

Iacovou, M., Kaminska, O. & Levy, H. (2012). *Using EU-SILC data for cross-national analysis: strengths, problems and recommendations.* ISER Working Paper Series, No. 2012-03, Institute for Social and Economic Research. <https://www.econstor.eu/bitstream/10419/65951/1/686613252.pdf>

Raghunathan, T. E. & Grizzle, J.E. (1995). A split questionnaire survey design. *Journal of the American Statistical Association,* 90(429), 54–63. <https://doi.org/10.1080/01621459.1995.1047648894:896-908>

Rendall, M.S., & Greulich, A. (2016). Multiple imputation for demographic hazard models with left-censored predictor variables: Application to employment duration and fertility in the EU-SILC. *Demographic Research,* 35(38), 1135–1148. <https://doi.org/10.4054/DemRes.2016.35.38>

**Supplementary Tables:**

Table S1: Comparison of characteristics of childless women aged 18-39, observed at different times of the survey

|  |  | *year t & year t+1, only co-residentially partnered, excl. still in education* | *year t-1, only co- residentially partnered, excl. still in education* | *year t-1, only co- residentially partnered, incl. still in education* | *year t-1 , incl. non- partnered, incl. still in education* |
| --- | --- | --- | --- | --- | --- |
| Full-time employed at time of survey | | 68.5% | 60.4% | 56.8% | 60.4% |
| Educational Attainment | |  |  |  |  |
|  | Low | 11.4% | 16.5% | 16.0% | 21.6% |
|  | Medium | 37.8% | 44.3% | 45.1% | 50.3% |
|  | High | 50.8% | 39.2% | 38.9% | 28.1% |
| Married |  | 58.1% | 53.9% | 51.8% | 13.6% |
| Age |  | 31.0 (median) | 30.8 (median) | 30.5 (median) | 26.8 (median) |
| N of person-year observations: | | 12,060 | 20,510 | 22,095 | 116,026 |

Data Source: EU-SILC, 24 European countries, 2004-2017.

Table S2: Logistic regression of first birth^a^ for partnered women aged 18 to 39 by couple combination of full-time, full-year employment

|  |  | M1 | M2 | M3 | M4 |
| --- | --- | --- | --- | --- | --- |
| Couple combination of full-time, full-year employed^b^: | |  |  |  |  |
|  | both full-time, full-year employed | *Ref.* | *Ref.* |  |  |
|  |  |  |  |  |  |
|  | only woman full-time, full-year employed | -0.217* | -0.227* |  |  |
|  |  | (0.0969) | (0.0954) |  |  |
|  | only male partner full-time, full-year employed | -0.340*** | -0.315*** |  |  |
|  |  | (0.0623) | (0.0617) |  |  |
|  | neither full-time, full-year employed | -0.470*** | -0.480*** |  |  |
|  |  | (0.0898) | (0.0896) |  |  |
| More detailed combinations of couple labor-market activity statuses: | |  |  |  |  |
|  | both full-time, full-year employed |  |  | *Ref.* | *Ref.* |
|  |  |  |  |  |  |
|  | both full-year employed, either or both part-time |  |  | -0.118 | -0.111 |
|  |  |  |  | (0.0936) | (0.0923) |
|  | woman full-time, full-year employed; male partner ever-unemployed or ever-inactive |  |  | -0.238† | -0.231† |
|  |  |  |  | (0.128) | (0.125) |
|  | male partner full-time, full-year employed; woman ever-unemployed |  |  | -0.364*** | -0.352*** |
|  |  |  |  | (0.0905) | (0.0896) |
|  | male partner full-time, full year employed; woman ever-inactive (never unemployed) |  |  | -0.775*** | -0.669*** |
|  |  |  |  | (0.143) | (0.142) |
|  | either or both ever-students |  |  | -0.468** | -0.514** |
|  |  |  |  | (0.162) | (0.162) |
|  | either woman or male partner full-time, full-year employed; the other changed activity status^c^ |  |  | -0.171 | -0.188 |
|  |  |  |  | (0.131) | (0.131) |
|  | any other combination of neither full-time, full-year employed |  |  | -0.449*** | -0.454*** |
|  |  |  |  | (0.0929) | (0.0925) |
| Married^c^ | | 0.793*** |  | 0.802*** |  |
|  |  | (0.0583) |  | (0.0584) |  |
| Age^c^ |  |  |  |  |  |
|  | 18-22 | *Ref.* | *Ref.* | *Ref.* | *Ref.* |
|  |  |  |  |  |  |
|  | 23-27 | -0.153 | -0.0588 | -0.170 | -0.0757 |
|  |  | (0.175) | (0.175) | (0.175) | (0.176) |
|  | 28-32 | -0.0451 | 0.141 | -0.0701 | 0.115 |
|  |  | (0.172) | (0.172) | (0.173) | (0.173) |
|  | 33-39 | -0.637*** | -0.383* | -0.657*** | -0.405* |
|  |  | (0.176) | (0.175) | (0.177) | (0.176) |
| Constant | | -1.792*** | -1.483*** | -1.784*** | -1.468*** |
|  |  | (0.223) | (0.223) | (0.224) | (0.224) |
| N of person-year observations: | | 12,060 | 12,060 | 12,060 | 12,060 |
| N of respondents: | | 8,386 | 8,386 | 8,386 | 8,386 |
| Pseudo R² | | 0.0415 | 0.0229 | 0.0430 | 0.0241 |

Data Source: EU-SILC, 24 European countries, 2004-2017.

*Notes:* Estimated coefficients with robust standard errors in parentheses; † *p*<0.10, * *p*<0.05, ** *p*<0.01, *** *p*<0.001; all models include country- and year-fixed effects, and standard errors adjust for clustering within couple in the case of multiple observations of first-birth exposure per couple.

1. First-birth exposure starts in Year t, and continues into Year t+1 (see Figure 1).
2. Full-time, full-year employment status for both the woman and her male partner is defined for the 12-month period two years (year_t-2_) before the year t that begins the year of exposure to first birth (see again Figure 1).
3. Age and marital status are defined at interview Year t.
